# A macrogenetic analysis of isolation mechanisms shaping genetic divergence in mammals

**DOI:** 10.1093/jhered/esag003

**Published:** 2026-01-21

**Authors:** Daniel A Hancock, Patrick Meirmans

**Affiliations:** Evolutionary and Population Biology, Institute for Biodiversity and Ecosystem Dynamics, University of Amsterdam, Amsterdam, The Netherlands; National Institute of Aquatic Resources, Technical University of Denmark, Silkeborg, Denmark; Evolutionary and Population Biology, Institute for Biodiversity and Ecosystem Dynamics, University of Amsterdam, Amsterdam, The Netherlands

**Keywords:** ecological niche modeling, isolation-by-distance, isolation-by-environment, isolation-by-resistance, landscape genetics, population structure

## Abstract

Understanding the processes that shape spatial genetic differentiation is essential for understanding how populations adapt to environmental change. By evaluating the influence of these processes, we can gain insights into evolutionary dynamics and the potential for species to respond to shifting landscapes. Three well-accepted processes that create spatial patterns in genetic variation are isolation-by-distance (IBD), where individuals are more genetically similar the closer they are geographically; isolation-by-environment (IBE), where gene flow is reduced due to selection against migrants in unsuitable ecological conditions; and isolation-by-resistance (IBR), where landscape features limit dispersal. We conducted a macrogenetic meta-analysis of single nucleotide polymorphism data to identify patterns shaping spatial genetic differentiation in 40 mammalian datasets globally. For each species, we built a species-distribution model and combined it with the global Human Footprint layer to create a composite resistance surface, revealing that habitat suitability and anthropogenic impact contribute roughly equally to resistance. Model selection tests found that IBR was the mechanism most frequently retained in the best models and had the highest variable importance. IBD and IBE alone had little effect but were often selected alongside IBR, suggesting that they may have secondary, context-dependent effects. However, the probability of finding IBD in the best model of divergence significantly increased as the number of populations sampled increased. Similarly, the probability of finding IBE increased with the spatial scale of the study. Our findings suggest that resistance is pervasive in shaping genetic variation in mammals worldwide, but that study design affects our ability to detect the presence of IBD and IBE.

## Introduction

Conserving biodiversity necessitates preserving evolutionary processes, which in turn requires an understanding of the mechanisms that shape population structure and gene flow to predict how species will adapt to future environmental changes (Moritz 1994; Frankham et al. 2010; Allendorf et al. 2012). When populations become isolated, they become more susceptible to genetic drift, resulting in the loss of genetic diversity and adaptive potential in the long term. In the short term, population isolation increases inbreeding and consequently inbreeding depression, which can negatively affect fitness (Hoffmann et al. 2017). Isolation of populations and genetic divergence between them can be shaped by several mechanisms, of which the three most well-studied include isolation-by-distance (IBD), isolation-by-environment (IBE), and isolation-by-resistance (IBR).

IBD (Wright 1943) represents one of the earliest formal population-genetic models of spatial structure, describing an increase in genetic distance with an increase in geographic distance. When long-distance dispersal between populations is limited or restricted by geography, localized genetic drift drives population divergence faster than gene flow can maintain metapopulation-wide variation (Slatkin 1993; Rousset 1997). This results in a pattern of spatial autocorrelation, where individuals that are near to each other are also more genetically similar. Previous research has revealed IBD to be a common occurrence in natural populations (Slatkin 1993; Meirmans 2012), which fits behavioral observations that individuals rarely disperse very far during their lifetime (Greenwood 1980). Nevertheless, the concept of a simple, straight-line distance that is an essential part of IBD ignores heterogeneity in environments and landscapes. Heterogeneity in environmental conditions is known to influence patterns of gene flow between populations, independent of geographic distance. Even though the inherent simplicity of the IBD model may not fully capture the complexities of genetic differentiation in natural populations (Jenkins et al. 2010), IBD serves as a useful null model against which alternative models of divergence can be evaluated.

IBE (Wang and Bradburd 2014) describes an alternative mechanism in which genetic distances are larger between populations in more divergent environments; a process that can eventually result in ecological speciation by divergent selection between environments (Hendry 2004; Rundle and Nosil 2005). The environmental variables considered when measuring IBE depend on the ecology and life history of the species being studied, but also on the availability of environmental data in the form of Geographic Information System layers—spatially explicit environmental raster data. For terrestrial species, variables typically include elevation, temperature, and precipitation (Bradburd et al. 2013; Wang 2013), though other abiotic or biotic variables have also been used such as vegetation density and soil type (Via and Hawthorne 2002). Irrespective of the variables used, IBE is likely to be the primary mechanism shaping divergence when environmental distances explain variation in genetic distances that cannot be explained by IBD.

Alternatively, IBR is based on the idea that different parts of the landscape differ in their conduciveness to gene flow. In this model, the connectivity among populations is determined by the overall resistance of the landscape that connects them. IBR is thus defined as the correlation of genetic and “resistance” distances, a graph theoretic distance metric based on circuit theory (McRae 2006). This metric models the landscape as an electrical circuit, assigning resistance values to various features and accounting for all possible pathways of movement. The distribution of suitable habitat is often used as a proxy for dispersal ability when constructing resistance surfaces (Milanesi et al. 2017; Kunz et al. 2021), because the factors that promote survival are assumed to also influence dispersal, and most species are unlikely to disperse across large areas of unsuitable habitats. Human-altered environments such as urban areas, roads, and agricultural fields may also act as strong barriers to dispersal, contributing substantial resistance and reducing gene flow (Kozakiewicz et al. 2019). Although both habitat suitability and anthropogenic disturbance are likely to shape patterns of resistance, their relative contributions remain unclear.

Although considerable research has focused on the effects of landscape, geography, and environment on genetic differentiation between populations within specific species, relatively few studies have investigated the broader processes shaping the spatial distribution of genetic variation across multiple species and spatial scales. One paper that did analyze data from multiple species (Jenkins et al. 2010), only focused on a single process, IBD, and found that the probability of finding significant IBD increases with the number of populations tested. Another study investigated the relative effect of IBD and IBE across species and taxonomic groups and found IBE to be more common than IBD (Sexton et al. 2014). However, those authors did not report the number of populations tested for each species and neither review tested other potential determinants of genetic differentiation, such as spatial scale (Kozakiewicz et al. 2019). Furthermore, both studies were based on literature reviews and therefore included studies that differ in the choice of genetic marker, environmental variables, and method for calculating or selecting the best-supported process, which makes comparisons among studies difficult. This variation limits the comparability of findings and highlights the need for a standardized, cross-species analytical framework.

The increased availability of genome-wide single nucleotide polymorphism (SNP) data from population genetic studies of non-model organisms over the last decade has made such a framework possible. Leveraging this, we adopt a macrogenetic approach (Leigh et al. 2021), re-analyzing publicly available genomic data from multiple species to perform a comparative multispecies, multiprocess (IBD, IBE, IBR) analysis to look for general patterns in spatial genetic differentiation in mammals. In doing so, the following research questions are addressed:

- Which process, IBD, IBE, or IBR, shows the strongest association with genetic differentiation across a broad set of mammalian species?
- Does habitat suitability or anthropogenic impact contribute more to IBR in shaping genetic divergence in mammals?
- Does the probability of IBD, IBE, or IBR being included in the best model of genetic divergence depend on spatial scale or the number of populations sampled?

## Methods

### Genetic data collection

Forty genomic datasets with individual-level SNP data were gathered for 37 mammalian species including one monotreme and six marsupials. Datasets were collected from the literature through the Web of Science by searching for “SNPs” or “single-nucleotide-polymorphisms” in the following categories: Evolutionary Biology, Ecology, Biology, Zoology, Biodiversity Conservation. This produced >5,000 hits which were then manually curated to find mammalian SNP datasets that satisfied the following criteria: the data must have a minimum of four a priori populations (defined by the authors prior to any genetic clustering), a minimum of three individuals per population, genotyped populations that are not invasive or translocated, and at least 50 km between the two most distant populations. The last criterion was selected to give an adequate number of cells to find resistance distances between all populations given the resolution of the habitat suitability models. For some datasets, populations were removed to fit the criteria. For two species, *Sus scrofa* (wild boar) and *Panthera tigris* (tiger) adequate data were available to conduct separate tests at the continental and regional levels. Additionally, *Canis lupus* (wolf) had separate datasets for populations in North America and Europe. Data were downloaded freely from online repositories or granted by the authors on request. See Table 1 for a full list of all datasets retrieved and their sources.

**Table 1:** Species included in the analysis, continent, number of populations sampled, maximum distance between furthest two populations, and summaries of the genetic data including the number of SNPs, observed heterozygosity (Ho), and overall *F*_ST_.

Species	Continent	No. populations	Maximum pairwise distance (km)	No. SNPs	Ho	*F*_ST_	Source
*Brachylagus idahoensis*	NA	14	546	50,605	0.423	0.052	Byer et al. (2021)
*Canis latrans*	NA	28	4,468	22,935	0.026	0.022	Heppenheimer et al. (2018)
*Canis lupus (Europe)*	EU	10	2,796	80,223	0.230	0.099	Stronen et al. (2013)
*Canis lupus (North America)*	NA	6	4,135	13,092	0.176	0.064	Schweizer et al. (2015)
*Castor fiber*	EU	7	6,409	307	0.091	0.662	Senn et al. (2014)
*Cervus elaphus*	EU	16	2,688	50,842	0.244	0.157	Carranza et al. (2024)
*Cynomys parvidens*	NA	7	95	3,550	0.204	0.368	Giglio et al. (2021)
*Dasyurus hallucatus*	AUS	23	3,132	12,963	0.055	0.401	von Takach et al. (2024)
*Dipodomys microps*	NA	25	998	42,891	0.432	0.078	Farleigh and Jezkova (2023)
*Felis silvestris S.*	EU	13	2,880	97	0.364	0.138	von Thaden et al. (2020)
*Gulo gulo*	EU	4	1,021	354	0.443	0.034	Ekblom et al. (2018)
*Holochilus sciureus*	SA	5	2,629	17,514	0.091	0.108	Prado et al. (2019)
*Lynx lynx*	EU	8	2,430	97	0.308	0.185	Förster et al. (2018)
*Lynx rufus*	NA	5	149	13,521	0.157	0.057	Kozakiewicz et al. (2019)
*Microtus agrestis*	EU	9	2,427	35,436	0.080	0.515	Fletcher et al. (2019)
*Microtus richardsoni*	NA	5	1,253	10,379,876	0.074	0.185	Duckett et al. (2023), Carstens (2023)
*Myodes glareolus*	EU	103	3,302	6,078	0.173	0.329	Marková et al. (2020)
*Myotis lucifugus*	NA	4	2,529	1,582	0.039	0.001	Donaldson et al. (2017)
*Ningaui timealeyi*	AUS	24	527	4,841	0.197	0.039	Skey et al. (2023), Shaw 2023
*Ochotona princeps*	NA	14	549	49,074	0.191	0.249	Schmidt et al. (2021)
*Ornithorhynchus anatinus*	AUS	4	2,735	136,267	0.325	0.241	Martin et al. (2018)
*Ovis dalli dalli*	NA	16	1,875	189	0.280	0.212	Roffler et al. (2016)
*Ovis nivicola*	ASIA	6	999	1,121	0.222	0.084	Dotsev et al. (2018)
*Panthera tigris (India)*	ASIA	4	2,403	6,445,422	0.187	0.142	Armstrong et al. (2021)
*Panthera tigris (Asia)*	ASIA	4	6,068	6,445,422	0.173	0.186	Armstrong et al. (2021)
*Peromyscus leucopus*	NA	23	161	14,930	0.109	0.052	Munshi-South et al. (2016)
*Petauroides volans*	AUS	10	168	18,808	0.104	0.204	Knipler et al. (2023)
*Phascolarctos cinereus*	AUS	31	2,105	4,796	0.185	0.206	Lott et al. (2022)
*Plecotus austriacus*	EU	10	1,512	6,061	0.218	0.047	Razgour et al. (2018)
*Pseudomys chapmani*	AUS	9	548	3,771	0.252	0.041	Skey et al. (2023), Shaw (2023)
*Pseudomys hermannsburgensis*	AUS	30	663	3,804	0.147	0.015	Skey et al. (2023), Shaw (2023)
*Puma concolor*	NA	4	857	16,285	0.283	0.124	Gustafson et al. (2022)
*Rupicapra rupicapra*	EU	6	690	20,907	0.080	0.033	Leugger et al. (2021)
*Sarcophilus harrisii*	AUS	9	268	18,962	0.160	0.061	Epstein et al. (2016)
*Sus scrofa (Europe)*	EU	15	3,538	49,803	0.191	0.124	Iacolina et al. (2016)
*Sus scrofa (Italy)*	EU	5	920	48,296	0.239	0.095	Scandura et al. (2022)
*Ursus americanus*	NA	18	5,993	29,213	0.096	0.110	Puckett et al. (2015)
*Ursus maritimus*	NA	12	3,043	13,488	0.186	0.024	Jensen et al. (2020)
*Vombatus ursinus*	AUS	7	848	28,082	0.159	0.152	Martin et al. (2019)
*Vulpes vulpes*	EU	30	5,592	15,003	0.228	0.063	McDevitt et al. (2022)

Source literature provided and dataset DOI if available. AUS—Australasia; EU—Europe; NA—North America.

The majority of SNP data were downloaded or received in the variant call format (vcf) and were already filtered except for *Ornithorhynchus anatinus*, *P. tigris*, and *Sarcophilus harrisii* which were filtered with VCFtools (v0.1.17; Danecek et al. 2011), and *Brachylagus idahoensis*, *Ningaui timealeyi*, *Pseudomys hermannsburgensis*, and *Pseudomys chapmani* which were filtered with the dartR package (v2.9.7; Gruber et al. 2018; Mijangos et al. 2022) in R (R version 4.2.2; R Core Team 2022). All filtering was performed according to the methods in the original manuscripts. Although variation in filtering criteria may contribute to variability between datasets, we do not expect it to generate systematic biases favoring any particular isolation mechanism, as filtering methods affect all pairwise *F*_ST_-estimates within a dataset similarly and therefore do not affect their correlation with other distance matrices. Smaller vcf files were loaded into R with the “pegas” package (Paradis 2010) and dartR files with the “dartR” package and transformed with “dplyr” (v1.0.9; Wickham et al. 2022) into numerical genotypes for calculating genetic distances with *hierfstat* (Goudet 2005). Several datasets were downloaded in STRUCTURE (Pritchard et al. 2000) format, or as comma-separated-values (.csv) and did not require any preprocessing.

### Occurrence and environmental data

Species occurrences for the 37 mammalian species, to be used for creating habitat suitability maps by Species Distribution Modeling, were retrieved via the species name keyword search in the Global Biodiversity Information Facility (GBIF). The GBIF occurrence data were cleaned by removing duplicates as well as removing fossil and living specimens, known managed, introduced, and invasive occurrence records, old occurrence records (before 1900), and records with poor coordinate precision or high uncertainty (>10 km). The occurrences were additionally cleaned with the R package CoordinateCleaner (Zizka et al. 2019) to remove points in the sea as well as those within 2 km of country centroids, capital centroids, zoos, museums, and other biodiversity institutes. The code for the data cleaning process as well as the species distribution modeling and the rest of the analysis can be found with the other R scripts on GitHub. In several cases, there were relatively few occurrences from GBIF so the coordinates from the study in which SNPs were retrieved were added to the occurrence records. The number of occurrences before and after cleaning and/or supplementation from the genomic study can be found in Supplementary Table S1, along with all GBIF DOIs for each species. The cleaned occurrence records were also visually inspected for anomalies, such as isolated occurrences far outside the known range of the species (e.g. Tigers in North America) or clearly misplaced records that passed filters, and if any were present these were manually removed.

Three sets of geospatial data were used as explanatory variables for Species Distribution Modeling: a set of bioclimatic variables and two sets of landscape variables covering human impact and fractional land cover (Table 2). Raster files for 19 bioclimatic variables were downloaded at 2.5 arc min resolution (~4.6 km^2^ at the equator) from WORLDCLIM (v2.1; Fick and Hijmans 2017). Elevation was excluded to avoid potential confounding, as it is used in WorldClim’s interpolation of bioclimatic variables. Landscape variables covered both human impact data at ~ 0.5 arc min resolution from the Human Footprint maps (Venter et al. 2016) and fractional land cover maps at ~ 0.05 arc min resolution from the 2019 Copernicus Global Land Cover Layers (Buchhorn et al. 2020). The “raster” package (Hijmans 2023) in R was used to reproject the Human Footprint maps from the Mollweide projection to the WGS84 longitude–latitude coordinate-reference-system and to aggregate the maps to match the bioclimatic maps’ resolution by computing the mean value of the aggregated cells. The Copernicus fractional land cover maps, already in WGS84, were similarly aggregated. The Human Footprint variables were assigned pressure scores according to their perceived pressure on wildlife in the original paper (Venter et al. 2016). During aggregation to 2.5 arc min, the average score of each grid cell represented the mean urban pressure within that cell. Table 2 details the pressure scores assigned to Human Footprint variables.

**Table 2:** Geospatial predictors used in species distribution modeling.

Geospatial variable	Source	Description
Bio1	WorldClim	Annual mean temperature (°C)
Bio2	WorldClim	Mean diurnal range (°C) (mean of monthly (max temp—min temp) in °C)
Bio4	WorldClim	Temperature seasonality (°C) (standard deviation ×100)`
Bio8	WorldClim	mean temperature of wettest quarter (°c)
Bio12	WorldClim	Annual precipitation
Bio15	WorldClim	Precipitation seasonality (mm) (coefficient of variation)
Bio18	WorldClim	Precipitation of warmest quarter (mm)
Built2009	Human Footprint Maps	Urban environments including buildings, pavement, and urban parks. Assigned pressure score of 10.
Croplands2005	Human Footprint Maps	Intensive agricultural lands for growing crops. Assigned pressure score of 7
Pasture2009	Human Footprint Maps	Grazing lands for domesticated herbivores. Pressure score between 0 and 4.
Grass Fractional Cover	Copernicus	Values between 0 and 100 indicating % of pixel that is covered by grass
Shrub Fractional Cover	Copernicus	Values between 0 and 100 indicating % of pixel covered by shrubbery
Tree Fractional Cover	Copernicus	Values between 0 and 100 indicating % of pixel covered by trees

**Table 3:** Results of linear mixed effects models showing AICc, ΔAICc, and marginal *R*^2^ (*R*^2^_m_) values for all models with ΔAICc ≤ 2 for each species.

Species	Model	AICc	ΔAICc	*R* ^2^_m_
*Brachylagus idahoensis*	IBR	−665.0	0.000	0.922
*Brachylagus idahoensis*	IBE + IBR	−663.9	1.080	0.924
*Brachylagus idahoensis*	IBD + IBR	−663.1	1.918	0.920
*Canis latrans*	IBD + IBE + IBR	−2,218.3	0.000	0.661
*Canis lupus (Europe)*	IBR	−248.6	0.000	0.943
*Canis lupus (Europe)*	IBE + IBR	−248.5	0.145	0.952
*Canis lupus (Europe)*	IBD + IBE + IBR	−246.9	1.731	0.959
*Canis lupus (North America)*	IBR	−66.6	0.000	0.924
*Canis lupus (North America)*	IBE + IBR	−65.5	1.086	0.940
*Castor fiber*	IBE	−35.2	0.000	0.087
*Cervus elaphus*	IBR	−578.3	0.000	0.743
*Cervus elaphus*	IBE + IBR	−578.0	0.383	0.759
*Cervus elaphus*	IBD + IBR	−576.7	1.685	0.760
*Cynomys parvidens*	IBR	−25.7	0.000	0.746
*Cynomys parvidens*	IBD	−23.9	1.841	0.722
*Dasyurus hallucatus*	IBD + IBE + IBR	−624.2	0.000	0.798
*Dipodomys microps*	IBE + IBR	−1,399.1	0.000	0.544
*Dipodomys microps*	IBR	−1,398.6	0.489	0.533
*Dipodomys microps*	IBD + IBE + IBR	−1,398.0	1.150	0.557
*Dipodomys microps*	IBD + IBR	−1,397.5	1.625	0.547
*Felis silvestris S.*	IBD + IBR	−236.7	0.000	0.841
*Felis silvestris S.*	IBR	−234.9	1.722	0.842
*Gulo gulo*	IBD + IBE + IBR	−131.6	0.000	0.992
*Holochilus sciureus*	IBE	−26.3	0.000	0.775
*Holochilus sciureus*	IBR	−25.9	0.339	0.729
*Lynx lynx*	IBR	−99.3	0.000	0.217
*Lynx rufus*	IBR	−43.9	0.000	0.846
*Microtus agrestis*	IBE + IBR	−75.7	0.000	0.423
*Microtus richardsoni*	IBR	−23.2	0.000	0.599
*Myodes glareolus*	IBE + IBR	−14,988.3	0.000	0.696
*Myodes glareolus*	IBD + IBE + IBR	−14,986.3	1.986	0.696
*Myotis lucifugus*	IBD + IBE + IBR	−161.5	0.000	0.880
*Ningaui timealeyi*	IBD + IBE + IBR	−1,547.2	0.000	0.473
*Ningaui timealeyi*	IBD + IBR	−1,546.0	1.222	0.460
*Ochotona princeps*	IBR	−348.7	0.000	0.942
*Ornithorhynchus anatinus*	IBD + IBE + IBR	−113.0	0.000	0.998
*Ovis dalli dalli*	IBD + IBR	−289.5	0.000	0.551
*Ovis dalli dalli*	IBD + IBE + IBR	−287.8	1.715	0.545
*Ovis nivicola*	IBD + IBR	−95.4	0.000	0.984
*Ovis nivicola*	IBD + IBE + IBR	−95.0	0.348	0.989
*Ovis nivicola*	IBR	−95.0	0.372	0.978
*Panthera tigris (Asia)*	IBD + IBE + IBR	−102.6	0.000	0.882
*Panthera tigris (India)*	IBD + IBE + IBR	−106.5	0.000	0.962
*Peromyscus leucopus*	IBR	−836.6	0.000	0.211
*Peromyscus leucopus*	IBE + IBR	−835.6	0.994	0.191
*Peromyscus leucopus*	IBD + IBE + IBR	−835.1	1.483	0.230
*Peromyscus leucopus*	IBD + IBR	−834.7	1.891	0.230
*Petauroides volans*	IBD	−174.1	0.000	0.521
*Phascolarctos cinereus*	IBD + IBE + IBR	−1,797.1	0.000	0.767
*Phascolarctos cinereus*	IBR	−1,796.5	0.652	0.771
*Phascolarctos cinereus*	IBD + IBR	−1,796.5	0.680	0.767
*Phascolarctos cinereus*	IBE + IBR	−1,795.9	1.241	0.772
*Plecotus austriacus*	IBR	−313.8	0.000	0.866
*Plecotus austriacus*	IBD + IBR	−312.4	1.484	0.850
*Pseudomys chapmani*	IBR	−258.9	0.000	0.043
*Pseudomys chapmani*	IBE + IBR	−257.1	1.803	0.061
*Pseudomys hermannsburgensis*	IBD + IBE + IBR	−338.1	0.000	0.395
*Pseudomys hermannsburgensis*	IBR	−337.6	0.566	0.236
*Pseudomys hermannsburgensis*	IBE + IBR	−337.1	0.991	0.306
*Pseudomys hermannsburgensis*	IBE	−336.5	1.601	0.080
*Puma concolor*	IBD + IBE + IBR	−128.3	0.000	0.997
*Rupicapra rupicapra*	IBE + IBR	−111.7	0.000	0.958
*Rupicapra rupicapra*	IBR	−111.5	0.222	0.942
*Sarcophilus harrisii*	IBR	−177.6	0.000	0.783
*Sarcophilus harrisii*	IBE + IBR	−175.6	1.939	0.786
*Sus scrofa (Europe)*	IBD + IBR	−576.5	0.000	0.641
*Sus scrofa (Europe)*	IBR	−576.4	0.153	0.599
*Sus scrofa (Europe)*	IBD + IBE + IBR	−576.2	0.295	0.635
*Sus scrofa (Europe)*	IBE + IBR	−575.9	0.604	0.590
*Sus scrofa (Italy)*	IBR	−24.7	0.000	0.369
*Ursus americanus*	IBD + IBE + IBR	−438.0	0.000	0.685
*Ursus americanus*	IBR	−437.5	0.453	0.679
*Ursus americanus*	IBD + IBR	−437.2	0.792	0.681
*Ursus americanus*	IBE + IBR	−436.8	1.205	0.681
*Ursus maritimus*	IBD	−385.8	0.000	0.568
*Ursus maritimus*	IBD + IBR	−385.6	0.239	0.511
*Vombatus ursinus*	IBR	−63.7	0.000	0.501
*Vulpes vulpes*	IBD + IBE + IBR	−4,351.0	0.000	0.787
*Vulpes vulpes*	IBE + IBR	−4,349.9	1.091	0.797

**Table 4:** Results of generalized dissimilarity modeling showing the predictors with nonzero I-spline coefficients retained in the final model, total deviance explained by the model and the variable importance (deviance explained) of each predictor per species.

Species	Model	Deviance	IBD_imp	IBE_imp	IBR_imp
*Brachylagus idahoensis*	IBD + IBR	85.38	0.20	NA	49.60
*Canis latrans*	IBD + IBE + IBR	41.22	3.64	3.24	4.80
*Canis lupus (Europe)*	IBD + IBE + IBR	96.33	0.75	5.17	71.33
*Canis lupus (North America)*	IBD + IBE + IBR	94.53	0.70	1.05	77.68
*Castor fiber*	IBD + IBE + IBR	40.14	3.22	7.96	7.89
*Cervus elaphus*	IBD + IBE + IBR	61.83	0.93	0.29	27.82
*Cynomys parvidens*	IBR	66.77	NA	NA	66.77
*Dasyurus hallucatus*	IBD + IBE + IBR	71.43	2.98	0.40	26.29
*Dipodomys microps*	IBD + IBR	41.58	0.23	NA	8.66
*Felis silvestris S.*	IBD + IBE + IBR	82.14	0.06	0.01	20.68
*Gulo gulo*	IBR	93.59	NA	NA	93.59
*Holochilus sciureus*	IBE + IBR	76.75	NA	24.02	16.88
*Lynx lynx*	IBD + IBE + IBR	71.37	0.31	0.55	39.78
*Lynx rufus*	IBR	83.11	NA	NA	83.11
*Microtus agrestis*	IBD + IBE + IBR	59.58	3.64	10.05	0.14
*Microtus richardsoni*	IBD + IBE + IBR	70.84	0.01	12.10	8.21
*Myodes glareolus*	IBD + IBE + IBR	39.47	1.20	1.57	14.57
*Myotis lucifugus*	IBR	16.59	NA	NA	16.59
*Ningaui timealeyi*	IBD	46.75	46.75	NA	NA
*Ochotona princeps*	IBE + IBR	90.24	NA	NA	51.94
*Ornithorhynchus anatinus*	IBD + IBR	99.88	0.00	NA	41.19
*Ovis dalli dalli*	IBD + IBE + IBR	43.54	4.09	0.40	21.94
*Ovis nivicola*	IBD + IBR	98.36	0.89	NA	13.27
*Panthera tigris (Asia)*	IBD + IBR	76.09	0.55	NA	71.03
*Panthera tigris (India)*	IBD	2.44	2.44	NA	NA
*Peromyscus leucopus*	IBD + IBR	13.04	0.88	NA	12.38
*Petauroides volans*	IBD + IBR	72.30	23.35	NA	8.46
*Phascolarctos cinereus*	IBD + IBR	52.27	6.93	NA	11.48
*Plecotus austriacus*	IBR	87.64	NA	NA	87.64
*Pseudomys chapmani*	IBD	14.86	14.86	NA	NA
*Pseudomys hermannsburgensis*	IBR	24.58	NA	NA	24.58
*Puma concolor*	IBR	97.75	NA	NA	97.75
*Rupicapra rupicapra*	IBD + IBE + IBR	93.45	2.08	0.42	24.41
*Sarcophilus harrisii*	IBE + IBR	67.49	NA	0.58	45.90
*Sus scrofa (Europe)*	IBR	34.35	NA	NA	34.35
*Sus scrofa (Italy)*	IBR	29.06	NA	NA	29.06
*Ursus americanus*	IBE + IBR	56.55	NA	0.00	29.17
*Ursus maritimus*	IBD + IBE	40.45	27.13	0.17	NA
*Vombatus ursinus*	IBD + IBR	42.16	1.28	NA	20.75
*Vulpes vulpes*	IBD + IBE + IBR	66.62	0.15	0.12	45.53

Prior to species distribution modeling, environmental variables were assessed for collinearity. To keep explanatory variables consistent across species, values were extracted at every coordinate from the cleaned GBIF occurrence records for all species globally, and correlation matrices were created from the extracted values. When the Pearson correlation coefficient between a pair of variables was >0.7, the one with a simpler biological interpretability was retained. Several additional landscape variables were removed, even though they did not display collinearity, including navigable waterways, railways, moss/lichen fractional cover, snow cover, and permanent/seasonal water cover. These variables added unnecessary complexity to the species distribution models, almost always having an exceptionally low or non-existent influence on the final model. We also removed the “HFP2009” Human Footprint map of the cumulative anthropogenic pressure on the environment, as this was added separately as a resistance surface at a later step. Additionally, we found a strong association of occurrence records to the roads variable, likely due to a sampling bias present in the GBIF occurrences to easily accessible locations near to public roads (Reddy and Dávalos 2003; Kadmon et al. 2004; Gutierrez-Velez and Wiese 2020). Therefore, we also removed roads to prevent this bias propagating into the habitat suitability maps; the final set of variables used in the study is listed in Table 2.

### Species distribution modeling

One habitat suitability map was modeled per species using all variables in Table 2. For each species, the processed environmental predictors were loaded as rasters and cropped to the maximum extent of the species’ occurrence records plus 10 degrees in all four cardinal directions to provide a range of environmental backgrounds for pseudoabsence selection. Due to the absence of an independent test set, the cleaned occurrence records for each species were split into outer training (70%) and evaluation (30%) sets by random sampling without replacement, following Guisan and Thuiller (2005). To reduce sampling bias, the data were then thinned by using the gridSample function from the package dismo (Hijmans et al. 2023), taking two occurrence records per half-degree grid cell for the training set and one occurrence per half-degree grid cell for the test set. The outer training set was then further divided into inner training (70%) and testing (30%) sets as suggested by Hao et al. (2019), independently drawn for each of five replicate runs for cross-validation during modeling.

The “biomod2” R-package (v4.2-5; Thuiller et al. 2024) was used for Species Distribution Modeling to create habitat suitability maps that were subsequently used as input to calculate resistance matrices. *Biomod2* offers 10 different species distribution modeling techniques and multiple pseudoabsence selection schemes; after initial testing of all 10 techniques, Random Forests and Boosted Regression Trees were found to have the best predictive performance on the outer evaluation set. This is possibly due to these algorithms being the best performers with default parameters in *biomod2*. Therefore, the pseudoabsence selection scheme was optimized for these two machine learning algorithms and only these were used for model building. Five pseudoabsence replicates per species were generated with the BIOMOD_FormattingData function in “biomod2,” using the Surface Range Envelope (SRE) option with a quantile of 0.1 with the same number of pseudoabsences as the number of occurrences. This method of pseudoabsence generation has been found to provide good predictive capability for machine learning techniques in species distribution modeling (Barbet-Massin et al. 2012). Once the data were formatted, Random Forests and Boosted Regression Trees models were trained with “bigboss” parameters, predefined by the “biomod2” development team for niche modeling, and internally cross-validated five times for each pseudoabsence replicate. The resulting models were evaluated by the area under the receiver operating characteristic curve (AUC) on the “outer” evaluation data, except for four species where there were insufficient occurrences for an “outer” split and models were instead evaluated on the “inner” test data. The best five runs across all 50 combinations of algorithm, pseudoabsence replicate, and cross-validation were combined into an ensemble model by weighted mean, where individual models were weighted by their AUC values. The ensemble model was then projected to create a raster map of habitat suitability. Grid cells with 0 suitability were replaced by the minimum suitability (greater than 0) to allow for a slim chance of long-distance dispersal across unsuitable habitat. All projected ensemble species distribution models can be seen in Supplementary Figure S6. Finally, variable importance was assessed with the built-in functionality in the “biomod2” R package, calculated by shuffling each one of the variables in turn and computing the correlation between the reference predictions and predictions from the shuffled data.

### Calculating and optimizing distances

Genetic distances were calculated as population-level pairwise *F*_ST_ estimates. For large vcf files, this was calculated with VCFTools, otherwise, all genetic distances were calculated with “hierfstat” in R. Both methods of *F*_ST_ estimation use the Weir & Cockerham estimator, so they should lead to identical results. *F*_ST_ was selected as an estimator of genetic divergence in favor of alternative statistics such as *F’*_ST_ or *D*_est_, as these latter statistics were specifically developed for use with multiallelic markers such as microsatellites and *F*_ST_ is better suited for SNPs as these are biallelic markers (Meirmans and Hedrick 2010). We also tested a linearized *F*_ST_ (*F*_ST_/(1−*F*_ST_); Rousset 1997) but found this to slightly reduce model fit on average. Observed heterozygosity and overall *F*_ST_ were similarly calculated in “hierfstat” and VCFTools for each species and can be found with the number of SNPs in Table 1.

To test for IBD, great-circle distances were calculated between population coordinates with the “pegas” (Paradis 2010) package in R. Where sample data had coordinate references for individuals instead of populations, the mean longitude and latitude were calculated per population. Note that population definitions in this study were always established a priori in the source papers, prior to any clustering. In a few cases, this resulted in a population coordinate in the ocean or sea. When this occurred, the coordinate was manually corrected to the closest landmass.

To test for IBE, environmental distances were calculated by extracting values for all 19 bioclimatic variables at each of the population coordinates and then performing principal component analysis (PCA). In contrast to feature selection of bioclimatic variables during species distribution modeling, PCA takes advantage of multicollinearity to produce a set of uncorrelated principal components, hence for this analysis all 19 bioclimatic variables were kept. Environmental distance between populations was calculated as the distance between site scores on the first three principal components that explained most of the variance.

Finally, to test for IBR, pairwise resistance distances were calculated using Circuitscape 4.0.5 (McRae 2006) based on composite resistance surfaces that combined habitat suitability and anthropogenic impact. Resistance surfaces were optimized by Circuitscape in Julia (Bezanson et al. 2017) using the MS_optim function from the ResistanceGA R package (Peterman 2018), which allows objective parameterization of resistance models using a genetic algorithm without a priori assumptions. Each composite surface was optimized for the default, log-likelihood objective function with two input layers: a habitat suitability map derived from the Species Distribution Models (SDMs), and the Human Footprint Map representing anthropogenic impact. Habitat suitability was constrained to monomolecular transformations, based on the assumption that more suitable habitat should consistently confer lower resistance to gene flow. In contrast, the human footprint map was allowed to take on any transformation, to accommodate the possibility of more complex relationships. This allows for scenarios where, for instance, intermediate levels of human impact may facilitate dispersal, whereas both low and high levels may restrict it. For species with island populations, we assigned cells in the sea the maximum value in the Human Footprint Map, to allow Circuitscape to calculate resistances between focal nodes, similar to setting ocean cells to the minimum suitability in the SDM-generated surfaces. In Circuitscape, population coordinates were used as focal nodes between which resistance distances were calculated. The percentage of contribution of each input layer into the final composite resistance surface is output automatically by ResistanceGA for further analysis.

### Modeling the contributions of IBD, IBE, and IBR

Two model selection methods were used to test for associations between the distance metrics and genetic distance for identifying patterns of IBD, IBE, and IBR. The first was linear mixed effects (LME) models which have been shown to outperform other methods of model selection in terms of accuracy for landscape genetics studies (Shirk et al. 2018). We implemented LME based on the maximum likelihood population effects (MLPE) model using the mlpe_rga function from the “resistanceGA” R package (Peterman 2018) with REML = FALSE and population included as a random effect. The MLPE model accounts for the nonindependence among pairs of population distances by specifying the covariance structure of the response matrix, in this case the genetic distance matrix (Clarke et al. 2002), enabling a more accurate inference about the fixed effects of IBD, IBE, and IBR. In Shirk et al. 2018, selection of the best-supported model was achieved using the Akaike information criterion (AIC; Akaike 1973). However, model selection based on the original AIC can be unreliable with small sample sizes (*n* < 25) and highly correlated surfaces (Winiarski et al. 2020). Therefore, we used the sample-size corrected AICc instead. Models were ranked based on their AICc, with the model showing the lowest AICc considered the best. Following standard procedures, models with ΔAICc ≤ 2 relative to the best model were regarded as equivalent to the best model (Burnham and Anderson 2002). We also report the marginal *R*^2^ (*R*^2^_m_) for models with ΔAICc ≤ 2 to assess the variance explained by the fixed effects alone, providing a complementary measure of model performance and explanatory power beyond relative model ranking. All continuous predictors were mean-centered and scaled to unit variance prior to fitting.

We also employed a second method, generalized dissimilarity modeling (Ferrier et al. 2007) to assess the importance of mechanisms driving population divergence without assuming linear relationships between predictors and genetic distances (Murray et al. 2019; Worth et al. 2021). We implemented generalized dissimilarity models (GDMs) with the *gdm* package (v1.6.0-7, Fitzpatrick et al. 2025) in R. During model fitting, predictor variables with I-spline coefficients that sum to 0 were automatically excluded, indicating no detectable relationship with genetic dissimilarity. Variable importance for the retained predictors was assessed using 100 permutations with the *gdm.varImp* function which outputs the percentage of change in deviance explained by the model fit with and without that predictor. To obtain a metric that is comparable across models and species, we converted each relative value to an absolute contribution expressed in percentage points of total deviance explained. We also report the total deviance explained by each model as a measure of explanatory power, allowing comparison of model performance across species to see how well the included predictors account for patterns of genetic divergence. For most species, explanatory distance matrices were input without transformation, as predictors are transformed during model fitting; however, for six species (*B. idahoensis*, *Myotis lucifugus, N. timealeyi*, *P. hermannsburgensis, Ursus maritimus*, *Vulpes vulpes*), the genetic distance response matrix was re-scaled to aid model convergence (Mokany et al. 2022).

Logistic regression was used to determine whether the probability of IBD, IBE, or IBR being included in the most informative model is influenced by spatial scale or the number of populations sampled. A binary response variable was created for each mechanism, with one indicating the mechanism was included in one of the best models with the lowest AICc (where models with ΔAICc ≤ 2 are considered equivalent). Those binary response variables were then used as dependent variables in a logistic regression model with either the spatial scale of the number of populations as the explanatory variable. These logistic models were fit using the “glm” function in R. The number of populations sampled for *Myodes glareolus* was an order of magnitude higher than most of the other datasets. Therefore, this data point was removed prior to the logistic test of the number of populations only. Furthermore, log-transforming the maximum distance between populations (log_10_) reduced right skew and resulted in a slight improvement in model fit when testing the effect of spatial scale. Predicted values and 95% confidence intervals were computed on the logit scale by taking the fitted value ±1.96 times its standard error using predict(..., se.fit = TRUE) in R and then transforming these limits to the probability scale for plotting. To further test whether the strength of each mechanism, expressed as the absolute variable importance from GDMs, also varied with spatial scale or sample size, we used the “lm” function in R, with variable importance as the response and either log-transformed distance or number of populations as the predictor, also excluding *M. glareolus* from the population test.

## Results

### Species distribution modeling and composite resistance surfaces

The ensemble SDMs displayed a good fit between the occurrences and the environmental predictors, with an average AUC on inner test data of 0.97 and on outer evaluation data of 0.93. Bioclimatic variables were generally more important than landscape variables in predicting species distribution, with the two most important both related to temperature: Annual Mean Temperature (BIO1) and Temperature seasonality (BIO4), followed by Precipitation Seasonality (BIO15) and Annual Precipitation (BIO12). Fractional Tree cover was the fifth most important variable and the vegetation-related landscape variables (Tree, Grass, and Shrub cover) all contributed more to the SDMs than the Human Footprint variables (Pasture, Croplands, Built). A full breakdown of the top five models by AUC included in the ensemble models and the resulting AUCs can be found in Supplementary Table S2, variable importances in Supplementary Figure S3 and Supplementary Table S4, and projections and approximate sampling locations in Supplementary Figure S6.

**Fig. 1:**
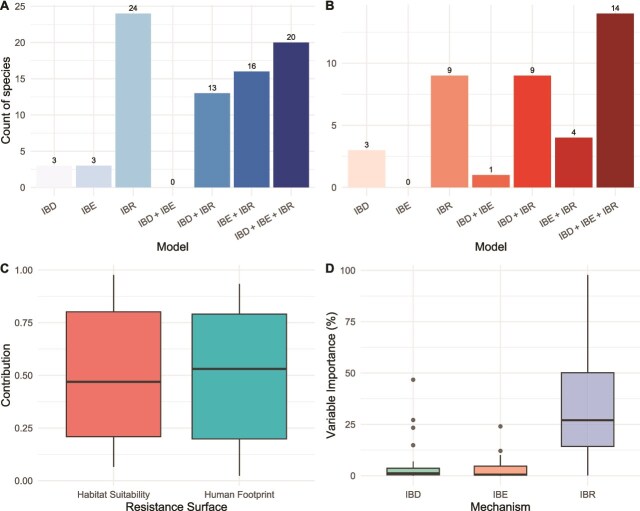
a) Count of datasets in which linear mixed effects (LME) models ranked within ΔAICc ≤ 2 of the best models. b) Count of datasets in which final generalized dissimilarity models (GDMs) included isolation mechanisms as predictors, where mechanisms that had no relationship to genetic dissimilarity were removed. c) The relative contribution of the habitat suitability layer and the Human Footprint layer to ResistanceGA (Peterman 2018) optimized composite resistance surfaces used to evaluate isolation-by-resistance (IBR). d) Variable importance of isolation‐by-distance (IBD), isolation-by-environment (IBE), and isolation-by-resistance (IBR) in the final GDMs. Importance is expressed as the percentage loss in total deviance explained when the predictor is permuted, that is, the relative loss returned by gdm.varImp multiplied by the model’s overall % deviance explained.

Combining the ensemble habitat-suitability layer together with the Human Footprint Map, ResistanceGA produced composite surfaces in which the two inputs contributed almost equally on average, with habitat suitability contributing 49.7% and human impact 50.3% (Fig. 1c). There were, however, large inter-specific differences: habitat suitability accounted for only 6% of the composite resistance surface in *S. scrofa* (within Italy), leaving human impact to explain the remaining 94%. Conversely, in *Ovis nivicola*, the pattern was reversed, with habitat suitability contributing 98% and human impact just 2%. The full set of species-specific % contributions is provided in Supplementary Table S5.

### Evaluating IBD, IBE, and IBR

When using LME to test for the relative importance of IBD, IBE, and IBR as models of genetic differentiation in mammals, IBR was most frequently included in the best-supported model (Table 3). IBR had the lowest or joint-lowest AICc in 24/40 datasets (Fig. 1a); in contrast, IBD and IBE alone only had the lowest or joint-lowest AICc in 3/40 datasets each. IBD and IBE were included in the best-supported model much more frequently when in combination with IBR (IBD + IBR = 13/40, IBE + IBR = 16/40, IBD + IBE + IBR = 20/40). Overall, models explained a substantial proportion of variance across species, with an average *R*^2^_m_ = 0.67 for all models with ΔAICc ≤ 2, although there was a large range in *R*^2^_m_ values, ranging from *R*^2^_m_ = 0.06 for *P. chapmani* to *R*^2^_m_ = 0.99 in *S. harrisii*. IBR alone explained more variance on average (*R*^2^_m_ = 0.67) than either IBD (*R*^2^_m_ = 0.60) or IBE (*R*^2^_m_ = 0.31) alone. Including IBR with one of the other two mechanisms yielded similar explanatory power to IBR alone (IBD + IBR: *R*^2^_m_ = 0.67, IBE + IBR: *R*^2^_m_ = 0.65). The highest variance explained was attained by models that included all three mechanisms (IBD + IBE + IBR: marginal *R*^2^_m_ = 0.74).

When the GDM approach was used, IBR was also most frequently included in the final model (Table 4), having nonzero I-spline coefficients in 36/40 datasets (Fig. 1b); much more often than either IBD (27/40) or IBE (19/40). In 9/40 datasets, IBR was the only predictor left with nonzero I-spline coefficients, whereas IBD was the only predictor left in just three datasets, and IBE was never the only predictor. Important to note is that a predictor being included in the GDM says nothing about the direction or strength of effect of that predictor, just that there was some relationship between the predictor and genetic distance, nor was there any model selection procedure as with LME. Indeed, the model including all predictors (IBD + IBE + IBR) was the most common outcome of generalized dissimilarity modeling, occurring in 14/40 datasets. Across all models, however, IBR contributed the most deviance, with an average absolute loss of 36% in explained deviance when it was permuted, versus only 6% for IBD and 4% for IBE (Fig. 1d). Overall, the GDMs explained 61.1% of the deviance in genetic distances on average, but this ranged from as low as just 2.4% for *P. tigris* (India) up to 99.9% in *S. harrisii.*

For the two species for which datasets were available at different spatial scales, there was rather good agreement as to the best model of genetic divergence between datasets. For the tiger (*P. tigris*), model selection with LME found IBD + IBE + IBR as the best model both at the smaller scale (India, *R*^2^_m_ = 0.96) and the larger scale (Asia, *R*^2^_m_ = 0.88). On the other hand, the GDM found nonzero I-spline coefficients for IBD + IBR at the larger scale, with a model total 76% deviance explained, 71% of which was due to IBR, whereas just 0.5% of it was due to IBD. At the smaller scale, the GDM found only IBD to have nonzero I-spline coefficients which only explained 2.4% of the total deviance. In the wild boar (*S. scrofa*) at the larger, Europe-wide scale, all the best models with ΔAICc ≤ 2 included IBR, with an average *R*^2^_m_ = 0.62. At the smaller scale, within Italy, only IBR was selected as the best model, with *R*^2^_m_ = 0.37. Similarly, in the GDMs, only IBR was retained at both spatial scales, with 34% and 29% deviance explained, respectively.

For the separate tests of wolf (*C. lupus*) populations in North America and Europe, there was also good agreement between tests. In North America, model selection with LME found IBR and IBE + IBR to be equally good at explaining genetic divergence (average *R*^2^_m_ = 0.93). In Europe, environment and resistance were also found to be important but with the addition of geographical distance, with IBR, IBE + IBR, and IBD + IBE + IBR all having ΔAICc ≤ 2 (average *R*^2^_m_ = 0.95). All three mechanisms were also kept in final GDMs for both datasets, with 94% deviance explained by the model in North American wolves and 96% in European wolves. In both cases, IBR had much higher variable importance, contributing 78% and 71% of the deviance in North America and Europe, respectively. IBE was second, contributing 5% and 1% and IBD third, contributing 0.75% and 0.7% to the deviance in North America and Europe respectively.

### The effect of sample size and spatial scale

**Fig. 2:**
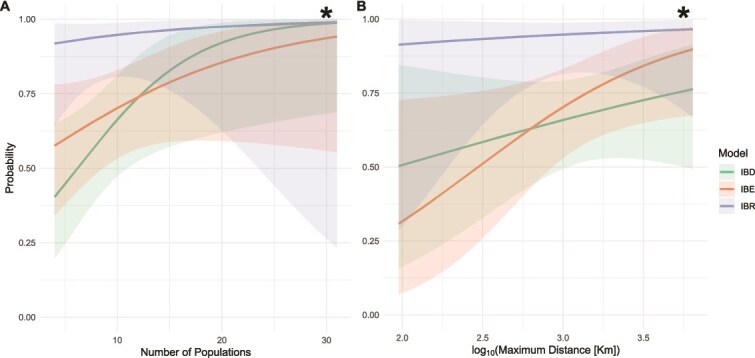
Logistic-regression probability curves (solid lines) with 95% confidence intervals (shaded) showing how the likelihood of each isolation mechanism being retained in the best Linear Mixed Effects model (ΔAICc ≤ 2) varies with (a) sample size, expressed as the number of populations, and (b) spatial scale, expressed as log₁₀ (maximum inter-population distance, km). Mechanisms: isolation-by-distance (IBD), isolation-by-environment (IBE), and isolation-by-resistance (IBR). ^*^a)—significant (*P* = 0.03) positive effect of population number on inclusion of IBD. ^*^b)—significant (*P* = 0.04) positive effect of log₁₀ (maximum distance) on inclusion of IBE.

**Fig. 3:**
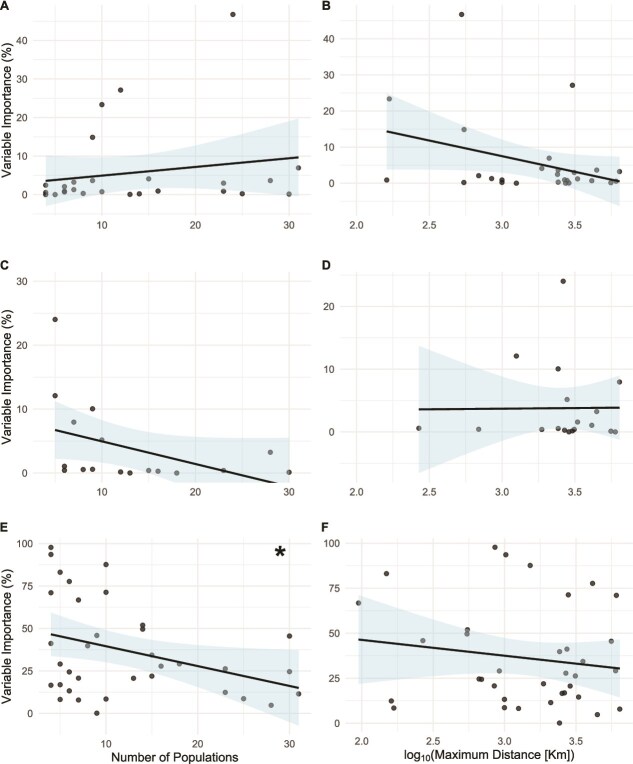
Variable importance (% deviance explained after permutation) of each isolation mechanism in Generalized Dissimilarity Models plotted against sampling attributes. Rows: isolation-by-distance (IBD, top), isolation-by-environment (IBE, middle), and isolation-by-resistance (IBR, bottom). Columns: left—number of populations sampled; right—spatial scale, log₁₀ (maximum inter-population distance, km). Points are individual datasets; lines are least-squares fits with 95% confidence ribbons. ^*^ Significant (*P* = 0.04) negative relationship between population number and IBR importance.

The number of populations sampled had a significant effect on the probability of IBD being included in the model selected by LME (β *=* 0.18, *P* = 0.03; Fig. 2a), datasets with more populations were more likely to retain IBD. No comparable effect was detected for IBE (β *=* 0.09, *P* = 0.12) or IBR (β *=* 0.08, *P* = 0.53). Although the variable importance of IBD in GDMs increased with sample size, the relationship did not reach the 5% significance threshold (β = 0.23, *P* = 0.38; Fig. 3a). Similarly, the relationship for IBE was also not significant (β = −0.35, *P =* 0.09; Fig. 3c). In contrast, IBR did show a significant negative relationship, with its importance declining as the sample size increased (β = −1.17, *P* = 0.03; Fig. 3e). Spatial scale (the maximum distance between the furthest two populations sampled) was positively associated with the probability of IBE being included in the best model selected by LME (β *=* 1.63, *P* = 0.04; Fig. 2b), but not with the inclusion of IBD (β *=* 0.63, *P* = 0.1) or IBR (β *=* 0.53, *P* = 0.8). Spatial scale had no significant effect on the variable importance of IBD (β *=* −8.66, *P* = 0.07; Fig. 3b), IBE (β *=* 0.20, *P* = 0.97; Fig. 3d), or IBR (β *=* −8.79, *P* = 0.37; Fig. 3f) in GDMs. Because IBD and IBE were retained in the final GDM for only 27 and 18 datasets, respectively (compared with 36 for IBR), the associated linear regression models were based on smaller sample sizes, which may have limited their power to detect significant relationships between variable importance of IBD or IBE and spatial scale or sample size.

## Discussion

Across the 40 datasets analyzed, IBR emerged as the dominant mechanism shaping spatial genetic divergence in mammals. IBR was most frequently included in the best model selected by AICc in LME models, and its variable importance values in the generalized dissimilarity models (GDMs) were considerably higher than those of IBD or IBE. IBR was selected as the best model of divergence, without the presence of IBD or IBE, in 60% of datasets tested, although other models including IBR with IBD and/or IBE often had ΔAICc ≤ 2 relative to the best model and so are equally valid models of divergence for that species. IBD and IBE were rarely selected alone as the best model of divergence but often included in models with IBR, suggesting they may exert secondary, context-dependent effects or that their detection may depend more on study design, such as the number of populations sampled or the spatial scale of the study.

The dominance of resistance in the best supported models has clear implications: human impact on the natural environment already has, and likely will continue to have, serious ramifications for genetic differentiation and isolation of mammal populations globally. We reveal a roughly equal contribution, on average, of habitat suitability and human impact to optimized composite resistance surfaces across the 40 datasets. Suitable habitat is projected to shift, shrink, or fragment across most terrestrial regions as the climate warms and precipitation regimes shift (IPCC 2022). As suitable habitat changes, landscapes that currently facilitate dispersal may become inhospitable, amplifying resistance and further isolating populations.

On the other hand, it is remarkable how important the cumulative human footprint is to resistance surfaces for species investigated here. This means that for many mammals the construction of roads, agricultural expansion, and urbanization has already strongly shaped spatial patterns of genetic diversity. We did not test whether anthropogenic impact increases or decreases connectivity and allowed for complex relationships between the optimized composite resistance surface and genetic distance. Therefore, it is possible that for some species, such as the red fox (*V. vulpes*), certain levels of human impact provide corridors for dispersal. On the other hand, urbanization in particular has been implicated in interrupting connectivity (Kozakiewicz et al. 2019) and is associated with reductions in genetic variation in mammal populations globally (Munshi-South et al. 2016; Schmidt et al. 2020; Schmidt et al. 2021). The convergence of ongoing land-use change with rapid climate-driven habitat shifts therefore likely poses a compounding threat.

Human-induced resistance may play an even more important role than the results suggest, as land-use change may be too recent to have a detectable effect in the genome (Landguth et al. 2010). In other words, populations that only recently became isolated due to anthropogenic habitat loss may not yet display strong genetic divergence, as insufficient time has elapsed for differences in allele frequencies to accumulate. Anthropogenic habitat degradation reduces effective population sizes and isolates remaining populations (Saunders et al. 1991), reducing gene flow which in turn reduces allelic diversity and heterozygosity in animals (Johnson and Munshi-South 2017). The combination of reduced effective population sizes, reduced gene flow, and therefore increased drift can lead to inbreeding depression and loss of adaptive capacity, negatively affecting population viability and significantly increasing the extinction risk in rapidly changing environments (Sgrò et al. 2011).

We also find that model selection was influenced by study design. The probability that IBE was included in the best LME model significantly increased with spatial scale. Similarly, the probability that IBD was included increased with the number of populations sampled. Because populations in most source studies were delineated operationally by sampling locality, variation in how populations are defined would directly affect the number of populations available for analysis and could influence the power to detect IBD. This is similar to the results of Jenkins et al. (2010) who found the probability of finding significant IBD increased with the number of populations tested. However, we deliberately avoided assessing the significance of finding IBD, IBE, or IBR as focusing on significance biases the estimates of effect sizes (Berner and Amrhein 2022). Instead, we evaluated the magnitude of influence of each mechanism using AICc-based model selection in the LME framework and variable importance in the GDMs.

In their meta-analysis, Jenkins et al. (2010) also found that the number of populations was inversely proportional to the predictive capability (*R*^2^) of IBD, suggesting that more complex nonlinear processes such as landscape heterogeneity contribute more to genetic divergence. However, in this study, we find that the importance of IBR significantly reduced as the number of populations increased. The generalized dissimilarity modelling approach that we used estimates the importance of the different distance metrics. Although GDMs showed a slight increase in the importance of IBD with increased sample size, this relationship did not reach significance, and both IBD and IBE were retained in fewer GDMs overall, so with reduced support relative to IBR. Therefore, the slight decline in IBR importance with sample size likely reflects an increased importance of IBD, despite the nonsignificant result. Therefore, our results show a consistent biological relevance of landscape resistance that is independent of study design.

Other meta-analyses across a wide taxonomic range have found IBE to be ubiquitous in nature (Shafer and Wolf 2013; Sexton et al. 2014). In contrast, our findings suggest that in mammals, environmental variation (IBE) on its own is rarely sufficient to explain patterns of genetic divergence. One explanation might be that mammals are phenotypically plastic, and genetically similar populations can survive in a wider range of environmental conditions than other taxa, resulting in weaker selection against migrants when dispersing into distinct environments. However, the frequent inclusion of IBE in the best additive models alongside IBD and IBR implies that environmental factors coincide with spatial and landscape features to influence gene flow. In other words, although ecological differences may not independently drive divergence, they may enhance or modify the effects of geographic or resistance distance in mammals.

The significant positive effect of spatial scale on IBE inclusion further suggests that environmental differences may simply be more important over larger scales, which would make sense, as environments are likely to be relatively similar over smaller scales. Therefore, IBE may appear weakly supported due to a bias in our dataset toward populations spread over smaller spatial scales. Furthermore, important environmental factors may have been overlooked in this study, as the importance of environmental factors is expected to differ among species depending on their niche and life history. Although we would expect that limitation to also affect the habitat suitability models and therefore the models including IBR.

Although study design influenced the probability of IBD or IBE being included in the best model, these effects do not undermine the pervasive support for IBR. In our LME model selection, only six out of the 79 best ranked models by AICc do not include IBR. Furthermore, four out of those six were ranked equally (ΔAICc ≤ 2) to IBR or models including IBR. Although the importance of IBR declined slightly as the number of populations increased—a pattern expected as IBD becomes more detectable—IBR remained included in nearly all best-supported models. These patterns indicate that, despite variation in sampling design, landscape resistance is a consistently influential mechanism shaping genetic divergence in mammals.

In general, the models fit well and explained a large proportion of the variance in genetic dissimilarity, with an average *R*^2^_m_ = 0.67 of the LME models with ΔAICc ≤ 2 and average deviance explained of 61% of the GDMs. Using both model selection methods increases confidence in the results, as for some species where one method showed a poor fit, the other method fit better. For example, the GDM for *P. tigris* (within India) had a deviance explained of just 2.4% but the LME model had *R*^2^_m_ = 0.96. Similarly, the deviance explained for *M. lucifugus* was just 16.6% but the LME model had *R*^2^_m_ = 0.88. Despite this, for some species, both methods had weak explanatory power such as for *P. chapmani*, which had an average *R*^2^_m_ = 0.05 for the LME model and 14.9% deviance explained by the GDM. This is possibly because we lacked specific variables that were important for that species, such as soil-moisture or aridity indices (Skey et al. 2023). We selected the environmental predictors for our SDMs as we hypothesized them to be important across many mammalian species at a fairly coarse resolution, so that they would capture the overall suitability of the landscape across long distances. Therefore, we acknowledge that the SDMs as we made them cannot be used for obtaining a detailed, fine-grained map of species occurrence for each species. In addition, other processes not included here shape spatial patterns of genetic differentiation, such as post-glacial recolonization and density-dependent dispersal, which could also reduce the variance explained for some datasets.

Another important consideration is that, although datasets were selected to consist only of nuclear SNPs, no distinction was made between neutral and nonneutral markers. This distinction could be important because loci affected by natural selection can show different patterns of population structure than neutral loci. The lack of support for IBE acting on its own, such as found in our study, is therefore not evidence of the lack of loci involved with local adaptation. Species with very high gene flow may have high divergence at only a few adaptive loci in a background of low divergence at all other loci (Milano et al. 2014). In such cases, the effects of IBE would only be visible at a relatively small proportion of the genome: at the adaptive loci and loci that are physically closely linked. Further research into the processes shaping genetic divergence in putatively adaptive loci, as determined by outlier tests or environmental association analyses, may yield results that are more informative for understanding local adaptation and the implications for conservation.

The species included in this study show a wide variety of life-history traits such as body size, lifespan, active time of day, and generation time. This could influence model selection, as species with longer generation times may not have detectable traces of recent habitat loss in the genome. Furthermore, our dataset contains mammals with very different dispersal strategies and habitat preferences, including open-area specialists, forest-dwellers, riparian mammals, one alpine species, and two bat species. This diversity in life histories, dispersal strategies, and habitat preferences introduces potential sources of confounding. However, the IBD, IBE, and IBR frameworks are general population-genetic and landscape-ecological concepts that should apply to any species whose dispersal and gene flow vary spatially. IBD arises whenever movement probability declines with distance, regardless of whether dispersal occurs through air, water, or across terrestrial surfaces. The bioclimatic variables used for IBE (temperature, precipitation, seasonality) influence all mammals via thermoregulatory demands, resource availability, and ecosystem productivity, and therefore remain biologically meaningful for riparian, alpine, semi-aquatic, and volant species. Furthermore, our IBR surfaces are derived from species-specific habitat suitability models combined with human impact, rather than generic terrestrial resistance layers. As a result, the resistance surfaces inherently reflect each species’ ecology. For example, trees emerged as one of the most important variables in determining habitat suitability in *M. lucifugus* bats, which means that areas with low resistance should have high tree cover and be suitable foraging and roosting environments. Therefore, although species differ in their habitat associations, these differences are incorporated into the resistance surfaces, and the consistency of our results across this ecological diversity suggests that the dominant patterns we identify emerge despite these varied ecological interactions.

Shirk, Landguth and Cushman (2018) found that violations of the assumption of linearity were influential in selecting the incorrect model, and transformations to improve the linearity are therefore often applied. Some nonlinear relationships were observed when plotting genetic distances against geographic, environmental, or resistance distances in this study. Most are likely explained by nonuniform sampling, with gaps between distant locations and environments creating nonlinear relationships that would likely become linear with more comprehensive sampling (Meirmans 2015). Additionally, species with island populations, such as the Platypus in Tasmania and various European species with populations in the British Isles, have high resistance to dispersal due to unsuitable habitats in the sea, contributing to nonlinear relationships. Because one of our two approaches, generalized dissimilarity modelling, explicitly accommodates nonlinear relationships, we chose not to transform the final distance matrices, and such transformations made little difference to the outcome when testing with LME models.

Landscape genetics studies have been transitioning from utilizing a handful of microsatellites to hundreds of thousands of SNPs over the last two decades (Hohenlohe et al. 2010). As the cost of SNP genotyping nonmodel organisms continues to drop and data are made openly available, it will become easier to perform multispecies landscape genomic studies to reveal general spatial patterns in neutral and even adaptive variation at higher resolution in ecologically relevant organisms. However, it will become infeasible to manually curate the quality of genomic data being fed into these models without the development of software that allows handling whole genome SNP datasets for multiple species at a time (Helyar et al. 2011). Furthermore, the relationships we found with geographical scale and the number of populations further illustrate that future population genomic studies should not only focus on having progressively more markers, but also on other equally—if not more— important considerations regarding sampling strategy and experimental design (Meirmans 2015).

## Supplementary Material

supplementary_information_esag003

## Data Availability

The data produced in this study (genetic, environmental and resistance distances) and the R code used to produce it, as well as for species distribution modelling and resistance surface optimization, are available on GitHub at https://github.com/dannyhancock/mammal-macrogenetics. The SNP and location data that were sourced from the literature can be accessed through the associated papers (see Table 1).
