# Supplementary material for: A macrogenetic analysis of isolation mechanisms shaping genetic divergence in mammals
Source: J Hered. 2026 Jan 21;117(4):656–71. doi: 10.1093/jhered/esag003 (PMC13326407; doi:10.1093/jhered/esag003)

**Supplemental Information for:**

**A Macrogenetic Analysis of Isolation Mechanisms Shaping Genetic Divergence in Mammals**

Daniel Hancock, Patrick Meirmans

**Table of Contents:**

| **GBIF Occurrences and DOIs** | Page 2 |
| --- | --- |
| **Ensemble Model Members and AUCs** | Page 5 |
| **Variable Importance of Ensemble SDMs (Figure)** | Page 6 |
| **Variable Importance of Ensemble SDMs (Table)** | Page 7 |
| **Contribution to Composite Resistance Surfaces** | Page 9 |
| **Ensemble Model Projections** | Page 10 |

1. **GBIF Occurrences and DOIs**

Table showing the number of GBIF occurrences per species before and after cleaning, if any individuals from the study were included in the model, the final number of occurrences used to build species distribution models and the date and doi of the GBIF download.

| **Species** | **GBIF Occurrence Records** | **Occurrences after cleaning** | **Additional occurrence coordinates from study** | **Final occurrences** | **GBIF download and doi** |
| --- | --- | --- | --- | --- | --- |
| *Brachylagus idahoensis* | 420 | 151 | 458 | 609 | GBIF.org (07 April 2022) GBIF Occurrence Download  https://doi.org/10.15468/dl.j2xwkp |
| *Canis latrans* | 41052 | 25664 | 0 | 25664 | GBIF.org (07 April 2022) GBIF Occurrence Download  https://doi.org/10.15468/dl.tjfkzr |
| *Canis lupus (Europe)* | 66405 | 15570 | 0 | 15570 | GBIF.org (07 April 2022) GBIF Occurrence Download  https://doi.org/10.15468/dl.x246us |
| *Canis lupus (North America)* | 11709 | 3799 | 0 | 3799 | GBIF.org (07 April 2022) GBIF Occurrence Download  https://doi.org/10.15468/dl.9e4p7m |
| *Castor fiber* | 184721 | 54104 | 0 | 54104 | GBIF.org (11 April 2022) GBIF Occurrence Download  https://doi.org/10.15468/dl.rmj5bm |
| *Cervus elaphus* | 546393 | 104019 | 0 | 104019 | GBIF.org (20 May 2025) GBIF Occurrence Download <https://doi.org/10.15468/dl.9a5wx3> |
| *Cynomys parvidens* | 267 | 156 | 7 | 163 | GBIF.org (07 April 2022) GBIF Occurrence Download  https://doi.org/10.15468/dl.sjdhbq |
| *Dasyurus hallucatus* | 12768 | 1243 | 0 | 1243 | GBIF.org (06 March 2024) GBIF Occurrence Download  https://doi.org/10.15468/dl.fz3vx5 |
| *Dipodomys microps* | 3948 | 976 | 0 | 976 | GBIF.org (20 May 2025) GBIF Occurrence Download <https://doi.org/10.15468/dl.k95ka7> |
| *Felis silvestris S.* | 26474 | 8465 | 0 | 8465 | GBIF.org (07 April 2022) GBIF Occurrence Download  https://doi.org/10.15468/dl.mhdakh |
| *Gulo gulo* | 22386 | 19092 | 0 | 19092 | GBIF.org (07 April 2022) GBIF Occurrence Download  https://doi.org/10.15468/dl.pk2kfq |
| *Holochilus sciureus* | 223 | 53 | 18 | 71 | GBIF.org (02 June 2022) GBIF Occurrence Download  https://doi.org/10.15468/dl.zvpxn7 |
| *Lynx lynx* | 58777 | 17855 | 0 | 17855 | GBIF.org (11 February 2025) GBIF Occurrence Download  https://doi.org/10.15468/dl.s5yr59 |
| *Lynx rufus* | 19229 | 10875 | 0 | 10875 | GBIF.org (06 April 2022) GBIF Occurrence Download  https://doi.org/10.15468/dl.qzz8hs |
| *Microtus agrestis* | 51395 | 16030 | 0 | 16030 | GBIF.org (08 April 2022) GBIF Occurrence Download  https://doi.org/10.15468/dl.vd43k8 |
| *Microtus richardsoni* | 1156 | 362 | 0 | 362 | GBIF.org (06 March 2024) GBIF Occurrence Download https://doi.org/10.15468/dl.p9jpb4 |
| *Myodes glareolus* | 111742 | 16903 | 0 | 16903 | GBIF.org (10 December 2021) GBIF Occurrence Download https://doi.org/10.15468/dl.u9qgut |
| *Myotis lucifugus* | 10640 | 2053 | 0 | 2053 | GBIF.org (07 April 2022) GBIF Occurrence Download  https://doi.org/10.15468/dl.d5uaw6 |
| *Ningaui timealeyi* | 769 | 309 | 0 | 309 | GBIF.org (06 March 2024) GBIF Occurrence Download  https://doi.org/10.15468/dl.pusvu6 |
| *Ochotona princeps* | 8509 | 4457 | 0 | 4457 | GBIF.org (02 June 2022) GBIF Occurrence Download  https://doi.org/10.15468/dl.unpu7n |
| *Ornithorhynchus anatinus* | 14836 | 7911 | 0 | 7911 | GBIF.org (4 July 2022) GBIF Occurrence Download https://doi.org/10.15468/dl.xe4yr5 |
| *Ovis dalli dalli* | 518 | 348 | 15 | 363 | GBIF.org (17 January 2025) GBIF Occurrence Download https://doi.org/10.15468/dl.ea9sq9 |
| *Ovis nivicola* | 81 | 14 | 39 | 53 | GBIF.org (07 April 2022) GBIF Occurrence Download  https://doi.org/10.15468/dl.gbf76z |
| *Panthera tigris* | 1021 | 285 | 0 | 285 | GBIF.org (5 July 2022) GBIF Occurrence Download https://doi.org/10.15468/dl.67mxt7 |
| *Peromyscus leucopus* | 68305 | 10475 | 0 | 10475 | GBIF.org (07 April 2022) GBIF Occurrence Download  https://doi.org/10.15468/dl.azbrcw |
| *Petauroides volans* | 31164 | 22921 | 0 | 22921 | GBIF.org (26 April 2024) GBIF Occurrence Download  https://doi.org/10.15468/dl.d7wm65 |
| *Phascolarctos cinereus* | 207520 | 130264 | 0 | 130264 | GBIF.org (26 April 2024) GBIF Occurrence Download https://doi.org/10.15468/dl.xrqqk6 |
| *Plecotus austriacus* | 11149 | 2783 | 0 | 2783 | GBIF.org (07 April 2022) GBIF Occurrence Download  https://doi.org/10.15468/dl.cwh8gf |
| *Pseudomys chapmani* | 218 | 86 | 74 | 160 | GBIF.org (06 March 2024) GBIF Occurrence Download  https://doi.org/10.15468/dl.fkms42 |
| *Pseudomys hermannsburgensis* | 12461 | 3334 | 0 | 3334 | GBIF.org (06 March 2024) GBIF Occurrence Download  https://doi.org/10.15468/dl.d4kv6c |
| *Puma concolor* | 5768 | 3184 | 0 | 3184 | GBIF.org (07 April 2022) GBIF Occurrence Download  https://doi.org/10.15468/dl.7kqwc9 |
| *Rupicapra rupicapra* | 71868 | 9994 | 0 | 9994 | GBIF.org (25 May 2022) GBIF Occurrence Download https://doi.org/10.15468/dl.qmhb4r |
| *Sarcophilus harrisii* | 25479 | 219 | 39 | 258 | GBIF.org (07 April 2022) GBIF Occurrence Download  https://doi.org/10.15468/dl.xmg9fe |
| *Sus scrofa* | 132564 | 51402 | 0 | 51402 | GBIF.org (07 April 2022) GBIF Occurrence Download  https://doi.org/10.15468/dl.ypvav7 |
| *Ursus americanus* | 19897 | 13060 | 0 | 13060 | GBIF.org (06 April 2022) GBIF Occurrence Download  https://doi.org/10.15468/dl.waqvey |
| *Ursus maritimus* | 3410 | 623 | 0 | 623 | GBIF.org (07 April 2022) GBIF Occurrence Download  https://doi.org/10.15468/dl.3a3gcx |
| *Vombatus ursinus* | 85527 | 21065 | 0 | 21065 | GBIF.org (07 April 2022) GBIF Occurrence Download  https://doi.org/10.15468/dl.6xp4j2 |
| *Vulpes vulpes* | 396360 | 116425 | 0 | 116425 | GBIF.org (07 April 2022) GBIF Occurrence Download  https://doi.org/10.15468/dl.7y5e3v |

1. **Ensemble Model Members and AUCs**

Table showing the results of species distribution modelling in biomod2 for each species. The top 5 model runs by AUC were included in the ensemble. The AUC of the ensemble model on ‘inner’ validation and ‘outer’ evaluation data are also shown here. RF – Random Forests, GBM - Boosted Regression Trees. NA indicates there were not enough occurrences for an outer evaluation set.

| **Species** | **first** | **second** | **third** | **fourth** | **fifth** | **AUC validation** | **AUC evaluation** |
| --- | --- | --- | --- | --- | --- | --- | --- |
| *Brachylagus idahoensis* | GBM | RF | GBM | RF | GBM | 0.974 | 0.938 |
| *Canis latrans* | RF | RF | RF | RF | RF | 0.945 | 0.897 |
| *Canis lupus (Europe)* | RF | RF | RF | RF | RF | 0.958 | 0.908 |
| *Canis lupus (North America)* | RF | RF | RF | RF | RF | 0.954 | 0.849 |
| *Castor fiber* | RF | RF | RF | RF | RF | 0.981 | 0.947 |
| *Cervus elaphus* | RF | RF | RF | RF | RF | 0.963 | 0.932 |
| *Cynomys parvidens* | RF | GBM | RF | GBM | RF | 0.997 | NA |
| *Dasyurus hallucatus* | RF | RF | GBM | GBM | GBM | 0.975 | 0.957 |
| *Dipodomys microps* | RF | RF | RF | RF | RF | 0.978 | 0.948 |
| *Felis silvestris S.* | RF | RF | RF | RF | RF | 0.969 | 0.929 |
| *Gulo gulo* | RF | RF | GBM | RF | GBM | 0.981 | 0.967 |
| *Holochilus sciureus* | RF | RF | RF | GBM | RF | 0.984 | NA |
| *Lynx lynx* | RF | RF | RF | RF | RF | 0.989 | 0.976 |
| *Lynx rufus* | RF | RF | RF | RF | RF | 0.958 | 0.887 |
| *Microtus agrestis* | RF | RF | RF | RF | RF | 0.955 | 0.872 |
| *Microtus richardsoni* | RF | RF | GBM | RF | RF | 0.973 | 0.984 |
| *Myodes glareolus* | RF | RF | RF | RF | RF | 0.955 | 0.901 |
| *Myotis lucifugus* | RF | RF | RF | RF | RF | 0.972 | 0.915 |
| *Ningaui timealeyi* | GBM | GBM | RF | RF | GBM | 0.976 | 0.983 |
| *Ochotona princeps* | RF | RF | RF | RF | RF | 0.981 | 0.953 |
| *Ornithorhynchus anatinus* | RF | RF | GBM | RF | RF | 0.971 | 0.962 |
| *Ovis dalli dalli* | RF | RF | GBM | GBM | RF | 0.965 | 0.911 |
| *Ovis nivicola* | RF | RF | GBM | GBM | RF | 0.969 | NA |
| *Panthera tigris* | RF | RF | RF | RF | RF | 0.986 | 0.941 |
| *Peromyscus leucopus* | RF | RF | RF | RF | RF | 0.96 | 0.904 |
| *Petauroides volans* | RF | GBM | RF | RF | GBM | 0.975 | 0.963 |
| *Phascolarctos cinereus* | RF | RF | RF | RF | RF | 0.96 | 0.933 |
| *Plecotus austriacus* | RF | RF | RF | RF | RF | 0.968 | 0.929 |
| *Pseudomys chapmani* | RF | RF | RF | RF | RF | 0.984 | NA |
| *Pseudomys hermannsburgensis* | RF | RF | RF | RF | RF | 0.962 | 0.908 |
| *Puma concolor* | RF | RF | RF | RF | RF | 0.966 | 0.922 |
| *Rupicapra rupicapra* | RF | RF | RF | RF | RF | 0.971 | 0.95 |
| *Sarcophilus harrisii* | GBM | GBM | GBM | GBM | GBM | 0.956 | 0.959 |
| *Sus scrofa* | RF | RF | RF | RF | RF | 0.973 | 0.946 |
| *Ursus Americanus* | RF | RF | RF | RF | RF | 0.968 | 0.92 |
| *Ursus maritimus* | GBM | GBM | GBM | RF | RF | 0.969 | 0.937 |
| *Vombatus ursinus* | RF | RF | RF | RF | RF | 0.978 | 0.939 |
| *Vulpes vulpes* | RF | RF | RF | RF | RF | 0.972 | 0.943 |

1. **Variable Importance of SDMs**

Boxplots showing the relative (proportional) importance of variables on the ensemble species distribution models.


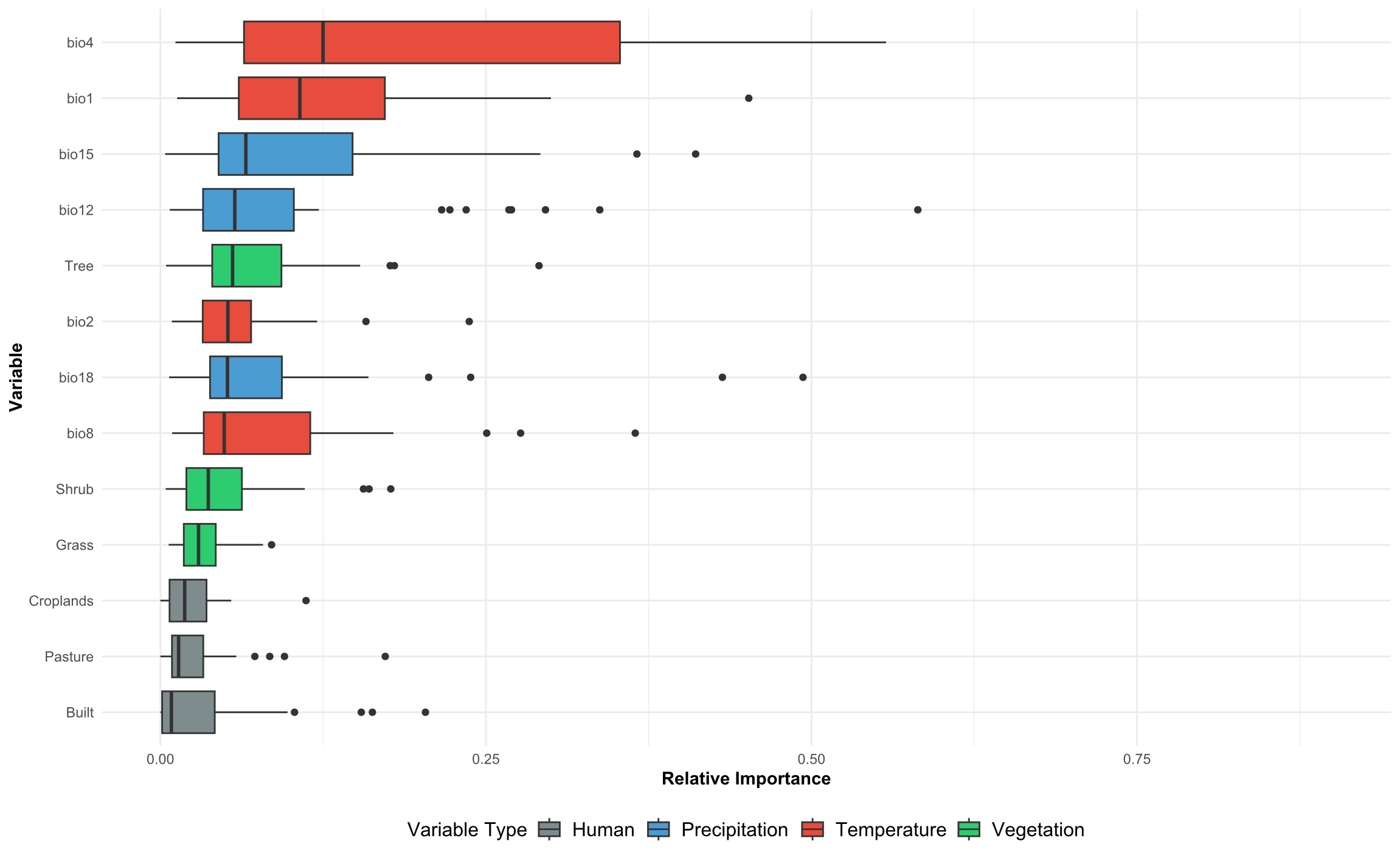


1. **Variable importance of SDMs**

Table showing the relative importance of each environmental variable in the habitat suitability models for each species. Bio1 = Annual Mean Temperature (°C), bio12 = Annual Precipitation, bio15 = Precipitation Seasonality (mm) (Coefficient of Variation), bio18 = Precipitation of Warmest Quarter (mm), bio2 = Mean Diurnal Range (°C) (Mean of monthly (max temp - min temp) in °C), bio4 = Temperature Seasonality (°C) (standard deviation ×100), bio8 = Mean Temperature of Wettest Quarter (°C).

| **Species** | **Built** | **Pasture** | **Croplands** | **Grass** | **Shrub** | **Tree** | **bio1** | **bio12** | **bio15** | **bio18** | **bio2** | **bio4** | **bio8** |
| --- | --- | --- | --- | --- | --- | --- | --- | --- | --- | --- | --- | --- | --- |
| *Brachylagus idahoensis* | 0.001 | 0.021 | 0.008 | 0.071 | 0.004 | 0.009 | 0.198 | 0.122 | 0.055 | 0.432 | 0.017 | 0.013 | 0.049 |
| *Canis latrans* | 0.154 | 0.034 | 0.029 | 0.043 | 0.052 | 0.070 | 0.230 | 0.040 | 0.048 | 0.045 | 0.098 | 0.107 | 0.049 |
| *Canis lupus (Europe)* | 0.053 | 0.016 | 0.035 | 0.021 | 0.019 | 0.076 | 0.053 | 0.094 | 0.065 | 0.073 | 0.052 | 0.404 | 0.038 |
| *Canis lupus (North America)* | 0.204 | 0.028 | 0.054 | 0.079 | 0.105 | 0.153 | 0.070 | 0.063 | 0.044 | 0.037 | 0.059 | 0.061 | 0.043 |
| *Castor fiber* | 0.034 | 0.018 | 0.048 | 0.046 | 0.016 | 0.134 | 0.119 | 0.071 | 0.118 | 0.160 | 0.060 | 0.143 | 0.031 |
| *Cervus elaphus* | 0.012 | 0.009 | 0.020 | 0.042 | 0.028 | 0.037 | 0.018 | 0.216 | 0.075 | 0.089 | 0.041 | 0.393 | 0.020 |
| *Cynomys parvidens* | 0.000 | 0.173 | 0.008 | 0.009 | 0.008 | 0.009 | 0.014 | 0.019 | 0.411 | 0.132 | 0.012 | 0.057 | 0.146 |
| *Dasyurus hallucatus* | 0.000 | 0.033 | 0.112 | 0.006 | 0.024 | 0.043 | 0.013 | 0.337 | 0.192 | 0.039 | 0.020 | 0.030 | 0.149 |
| *Dipodomys microps* | 0.001 | 0.058 | 0.002 | 0.038 | 0.017 | 0.040 | 0.063 | 0.085 | 0.225 | 0.206 | 0.082 | 0.125 | 0.059 |
| *Felis silvestris S.* | 0.015 | 0.014 | 0.036 | 0.024 | 0.038 | 0.041 | 0.103 | 0.064 | 0.078 | 0.049 | 0.120 | 0.380 | 0.037 |
| *Gulo gulo* | 0.004 | 0.000 | 0.013 | 0.015 | 0.061 | 0.004 | 0.058 | 0.024 | 0.009 | 0.014 | 0.009 | 0.513 | 0.277 |
| *Holochilus sciureus* | 0.035 | 0.004 | 0.009 | 0.074 | 0.043 | 0.052 | 0.040 | 0.012 | 0.292 | 0.015 | 0.158 | 0.016 | 0.251 |
| *Lynx lynx* | 0.008 | 0.008 | 0.024 | 0.011 | 0.012 | 0.069 | 0.084 | 0.031 | 0.186 | 0.057 | 0.070 | 0.398 | 0.041 |
| *Lynx rufus* | 0.091 | 0.033 | 0.041 | 0.040 | 0.059 | 0.108 | 0.223 | 0.036 | 0.035 | 0.070 | 0.052 | 0.172 | 0.040 |
| *Microtus agrestis* | 0.103 | 0.008 | 0.039 | 0.026 | 0.027 | 0.035 | 0.055 | 0.063 | 0.066 | 0.052 | 0.045 | 0.415 | 0.065 |
| *Microtus richardsoni* | 0.009 | 0.011 | 0.002 | 0.017 | 0.023 | 0.040 | 0.136 | 0.033 | 0.066 | 0.098 | 0.064 | 0.352 | 0.148 |
| *Myodes glareolus* | 0.090 | 0.013 | 0.037 | 0.025 | 0.038 | 0.176 | 0.066 | 0.087 | 0.073 | 0.088 | 0.071 | 0.210 | 0.024 |
| *Myotis lucifugus* | 0.163 | 0.009 | 0.017 | 0.036 | 0.076 | 0.139 | 0.288 | 0.030 | 0.031 | 0.045 | 0.031 | 0.068 | 0.069 |
| *Ningaui timealeyi* | 0.000 | 0.048 | 0.000 | 0.018 | 0.111 | 0.041 | 0.017 | 0.034 | 0.157 | 0.494 | 0.034 | 0.020 | 0.025 |
| *Ochotona princeps* | 0.002 | 0.010 | 0.003 | 0.021 | 0.026 | 0.041 | 0.300 | 0.032 | 0.048 | 0.030 | 0.043 | 0.265 | 0.178 |
| *Ornithorhynchus anatinus* | 0.006 | 0.012 | 0.012 | 0.019 | 0.064 | 0.066 | 0.107 | 0.111 | 0.060 | 0.063 | 0.045 | 0.070 | 0.365 |
| *Ovis dalli dalli* | 0.004 | 0.000 | 0.000 | 0.085 | 0.160 | 0.100 | 0.058 | 0.045 | 0.147 | 0.054 | 0.110 | 0.191 | 0.047 |
| *Ovis nivicola* | 0.000 | 0.001 | 0.000 | 0.053 | 0.033 | 0.055 | 0.452 | 0.070 | 0.045 | 0.125 | 0.009 | 0.012 | 0.145 |
| *Panthera tigris (Asia)* | 0.007 | 0.039 | 0.019 | 0.012 | 0.009 | 0.075 | 0.107 | 0.270 | 0.275 | 0.012 | 0.034 | 0.120 | 0.023 |
| *Panthera tigris (India)* | 0.001 | 0.011 | 0.010 | 0.035 | 0.010 | 0.180 | 0.148 | 0.022 | 0.366 | 0.007 | 0.075 | 0.120 | 0.017 |
| *Peromyscus leucopus* | 0.069 | 0.014 | 0.022 | 0.029 | 0.043 | 0.049 | 0.255 | 0.055 | 0.073 | 0.107 | 0.031 | 0.074 | 0.179 |
| *Petauroides volans* | 0.006 | 0.007 | 0.002 | 0.015 | 0.079 | 0.068 | 0.068 | 0.582 | 0.006 | 0.029 | 0.030 | 0.032 | 0.077 |
| *Phascolarctos cinereus* | 0.004 | 0.043 | 0.020 | 0.025 | 0.156 | 0.106 | 0.126 | 0.222 | 0.052 | 0.075 | 0.040 | 0.051 | 0.080 |
| *Plecotus austriacus* | 0.026 | 0.010 | 0.017 | 0.014 | 0.018 | 0.026 | 0.153 | 0.042 | 0.054 | 0.028 | 0.052 | 0.537 | 0.022 |
| *Pseudomys chapmani* | 0.000 | 0.095 | 0.000 | 0.035 | 0.177 | 0.018 | 0.037 | 0.026 | 0.237 | 0.238 | 0.017 | 0.076 | 0.042 |
| *Pseudomys hermannsburgensis* | 0.000 | 0.073 | 0.052 | 0.040 | 0.080 | 0.055 | 0.116 | 0.296 | 0.033 | 0.049 | 0.053 | 0.069 | 0.085 |
| *Puma concolor* | 0.007 | 0.018 | 0.018 | 0.022 | 0.037 | 0.072 | 0.151 | 0.026 | 0.123 | 0.069 | 0.069 | 0.354 | 0.034 |
| *Rupicapra rupicapra* | 0.012 | 0.022 | 0.025 | 0.042 | 0.041 | 0.075 | 0.118 | 0.235 | 0.040 | 0.102 | 0.053 | 0.201 | 0.034 |
| *Sarcophilus harrisii* | 0.000 | 0.005 | 0.006 | 0.011 | 0.090 | 0.005 | 0.284 | 0.007 | 0.004 | 0.011 | 0.011 | 0.557 | 0.009 |
| *Sus scrofa (Europe)* | 0.041 | 0.019 | 0.047 | 0.030 | 0.017 | 0.147 | 0.107 | 0.057 | 0.060 | 0.041 | 0.036 | 0.369 | 0.028 |
| *Ursus americanus* | 0.037 | 0.011 | 0.032 | 0.047 | 0.061 | 0.291 | 0.220 | 0.051 | 0.031 | 0.043 | 0.051 | 0.075 | 0.050 |
| *Ursus maritimus* | 0.043 | 0.000 | 0.000 | 0.064 | 0.020 | 0.029 | 0.087 | 0.037 | 0.148 | 0.026 | 0.237 | 0.276 | 0.032 |
| *Vombatus ursinus* | 0.003 | 0.084 | 0.031 | 0.018 | 0.026 | 0.046 | 0.191 | 0.268 | 0.042 | 0.050 | 0.052 | 0.020 | 0.167 |
| *Vulpes vulpes* | 0.098 | 0.014 | 0.037 | 0.054 | 0.036 | 0.086 | 0.063 | 0.077 | 0.100 | 0.045 | 0.071 | 0.259 | 0.060 |

1. **Contribution to Composite Resistance Surface**

Table shows the relative contributions of habitat suitability (from species distribution models) and cumulative human impact (from the Human Footprint Map) to the final resistance surfaces optimised in ResistanceGA.

| **Species** | **Habitat Suitability** | **Human Footprint Map** |
| --- | --- | --- |
| *Brachylagus idahoensis* | 0.84 | 0.16 |
| *Canis latrans* | 0.33 | 0.67 |
| *Canis lupus (Europe)* | 0.26 | 0.74 |
| *Canis lupus (North America)* | 0.65 | 0.35 |
| *Castor fiber* | 0.29 | 0.71 |
| *Cervus elaphus* | 0.63 | 0.37 |
| *Cynomys parvidens* | 0.94 | 0.06 |
| *Dasyurus hallucatus* | 0.54 | 0.46 |
| *Dipodomys microps* | 0.40 | 0.60 |
| *Felis silvestris S.* | 0.21 | 0.79 |
| *Gulo gulo* | 0.18 | 0.82 |
| *Holochilus sciureus* | 0.54 | 0.46 |
| *Lynx lynx* | 0.09 | 0.91 |
| *Lynx rufus* | 0.91 | 0.09 |
| *Microtus agrestis* | 0.28 | 0.72 |
| *Microtus richardsoni* | 0.91 | 0.09 |
| *Myodes glareolus* | 0.39 | 0.61 |
| *Myotis lucifugus* | 0.87 | 0.13 |
| *Ningaui timealeyi* | 0.73 | 0.27 |
| *Ochotona princeps* | 0.82 | 0.18 |
| *Ornithorhynchus anatinus* | 0.44 | 0.56 |
| *Ovis dalli dalli* | 0.67 | 0.33 |
| *Ovis nivicola* | 0.98 | 0.02 |
| *Panthera tigris (Asia)* | 0.91 | 0.09 |
| *Panthera tigris (India)* | 0.58 | 0.42 |
| *Peromyscus leucopus* | 0.08 | 0.92 |
| *Petauroides volans* | 0.89 | 0.11 |
| *Phascolarctos cinereus* | 0.78 | 0.22 |
| *Plecotus austriacus* | 0.24 | 0.76 |
| *Pseudomys chapmani* | 0.59 | 0.41 |
| *Pseudomys hermannsburgensis* | 0.12 | 0.88 |
| *Puma concolor* | 0.16 | 0.84 |
| *Rupicapra rupicapra* | 0.20 | 0.80 |
| *Sarcophilus harrisii* | 0.19 | 0.81 |
| *Sus scrofa (Europe)* | 0.47 | 0.53 |
| *Sus scrofa (Italy)* | 0.07 | 0.93 |
| *Ursus Americanus* | 0.84 | 0.16 |
| *Ursus maritimus* | 0.17 | 0.83 |
| *Vombatus ursinus* | 0.19 | 0.81 |
| *Vulpes vulpes* | 0.42 | 0.58 |

1. **Ensemble Model Projections**

Projections of species distribution models (SDMs) used as resistance surfaces for calculating resistance distances. Blue points are approximate population sampling locations. Some points are population averages of individually sampled coordinates, others may be approximated due to manual curation during processing for modelling and some authors requested that exact coordinate locations were not revealed.


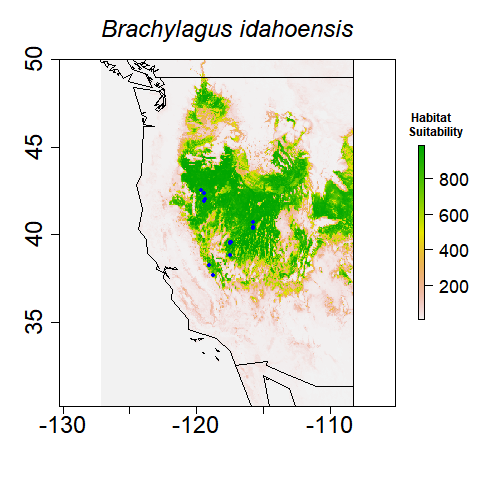

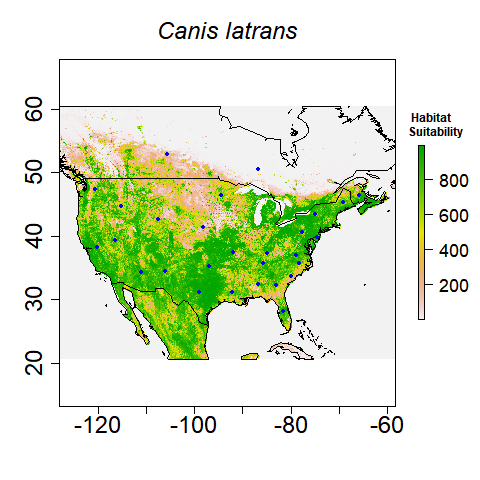

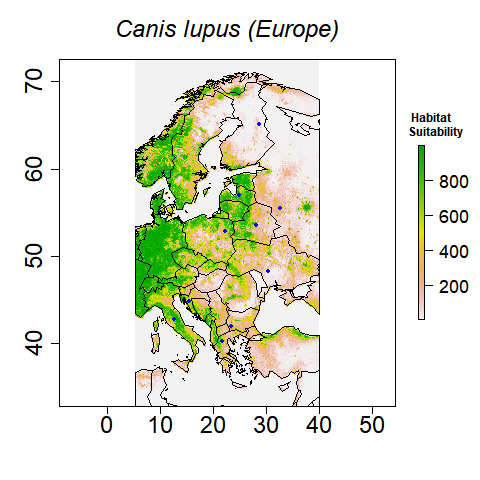

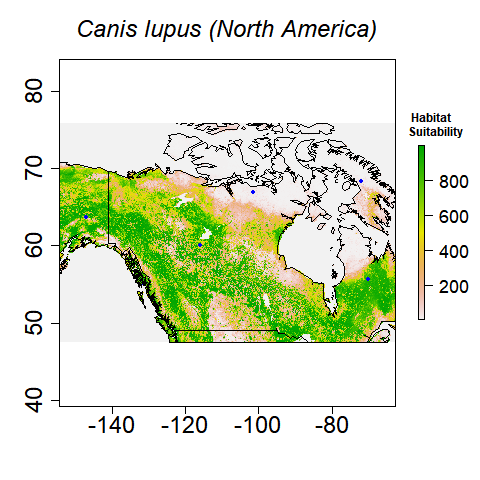

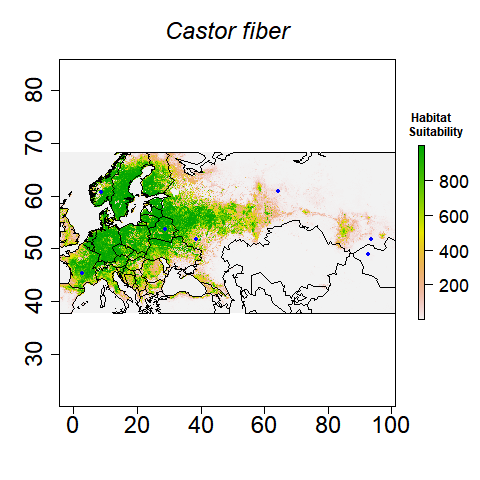

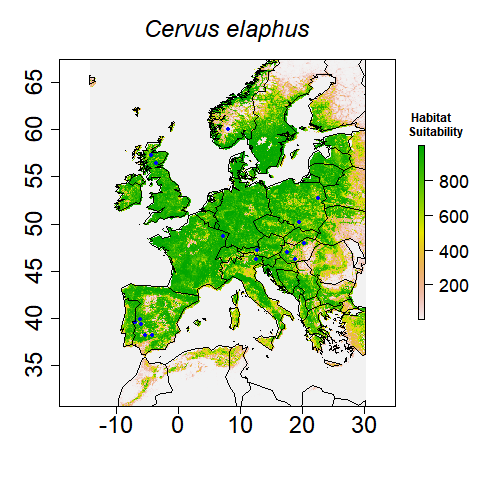

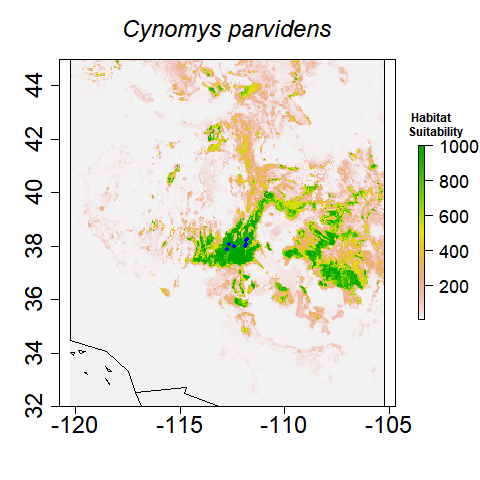

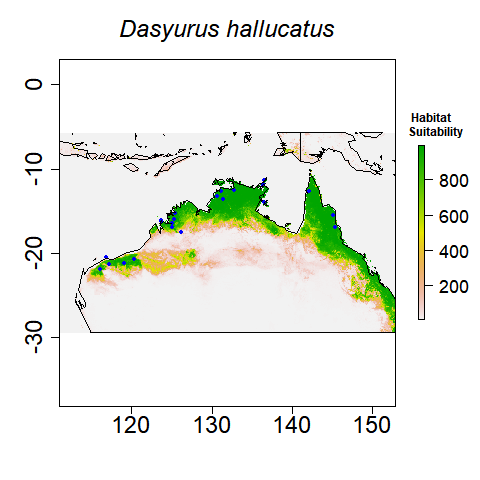

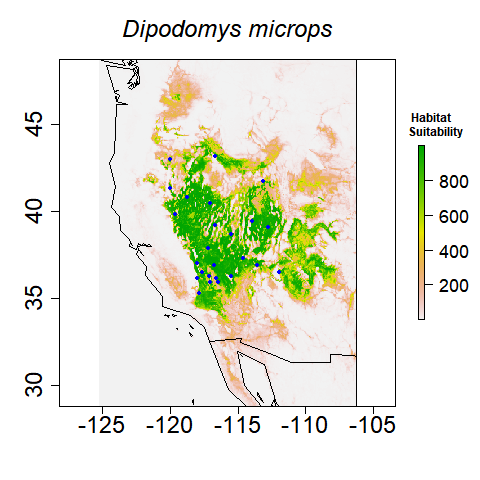

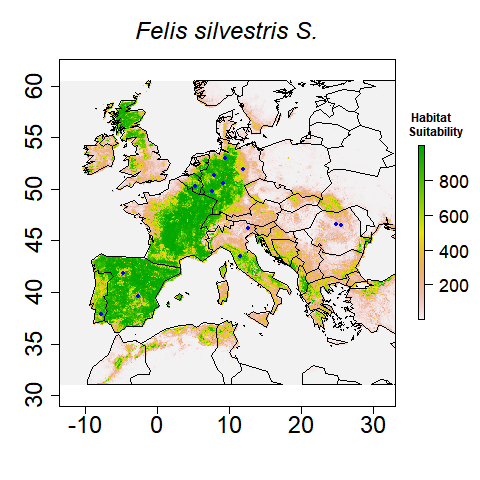

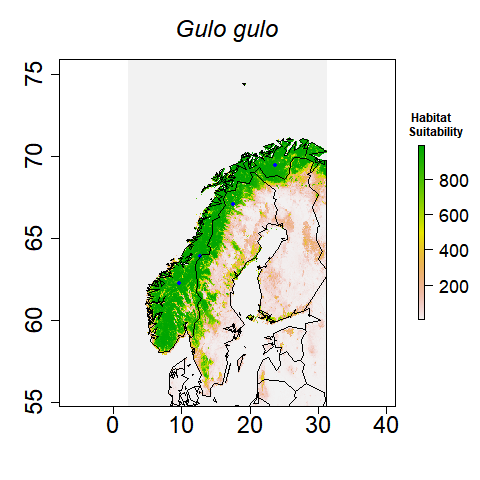

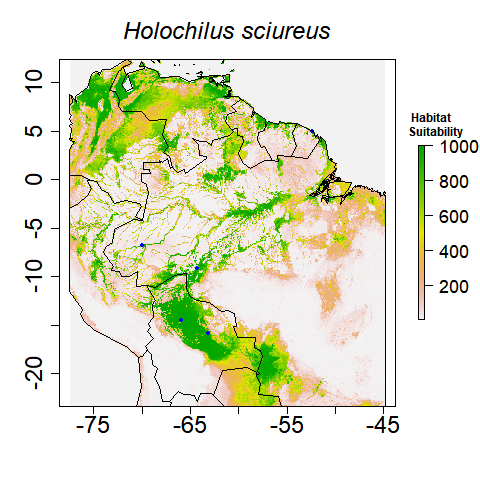

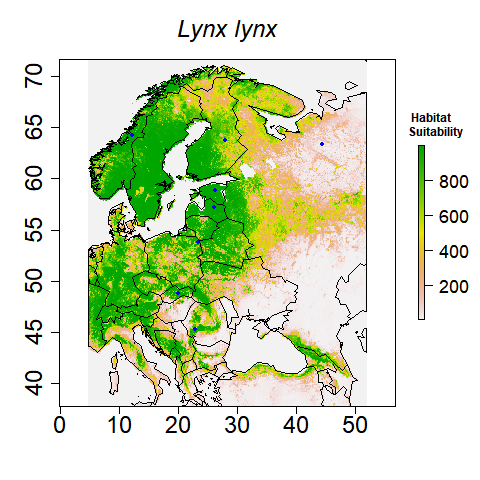

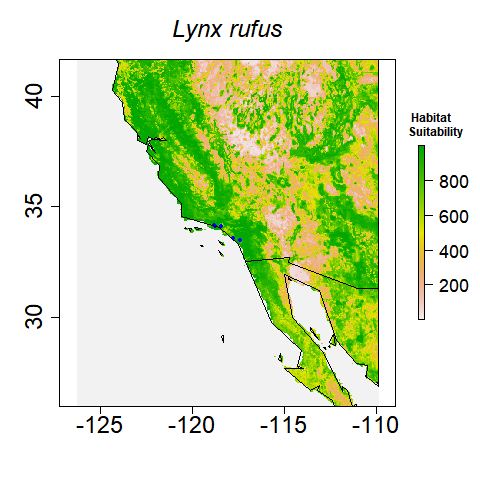

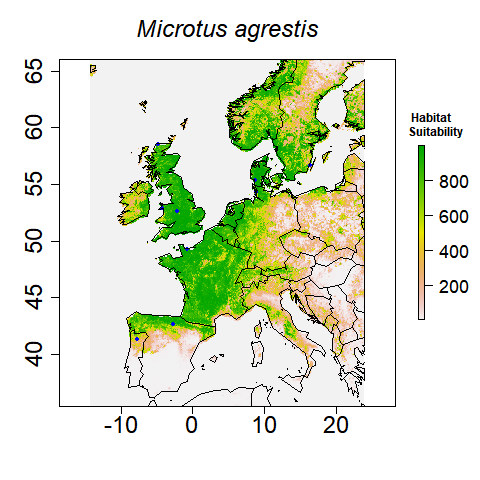

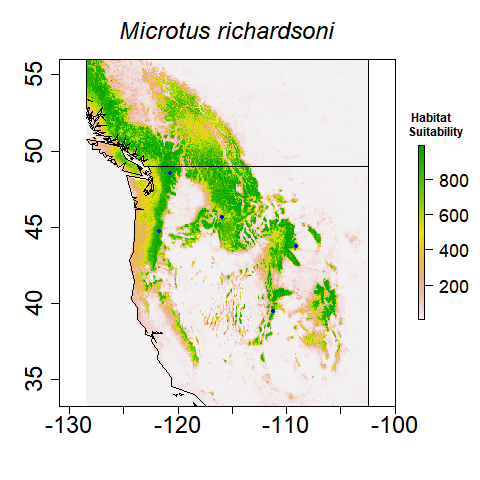

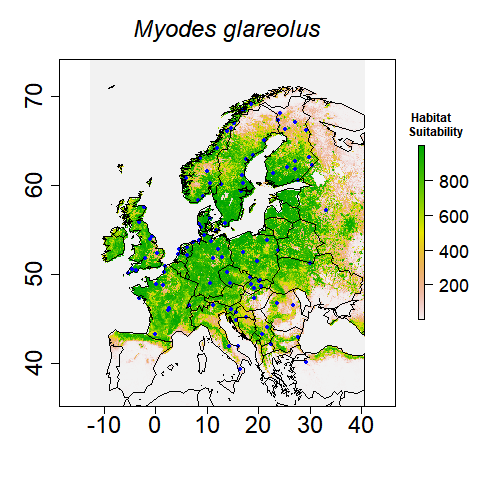

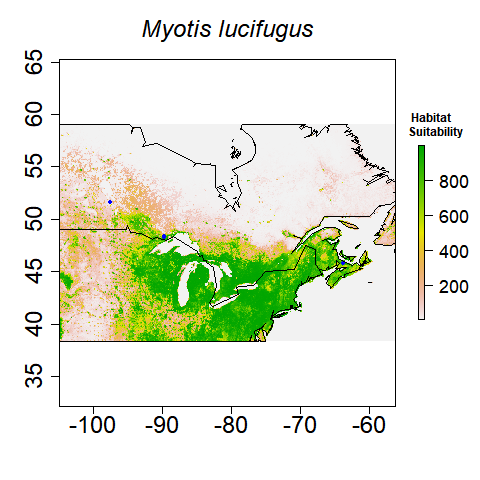

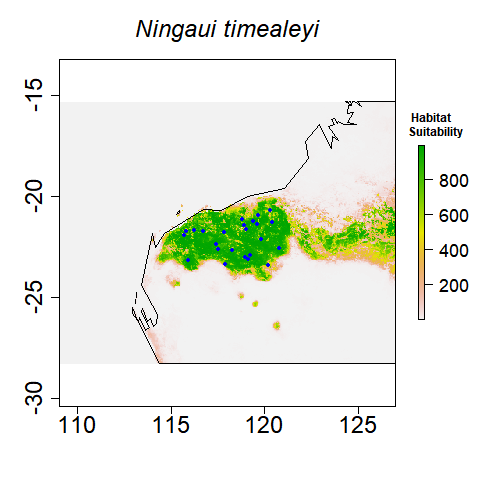

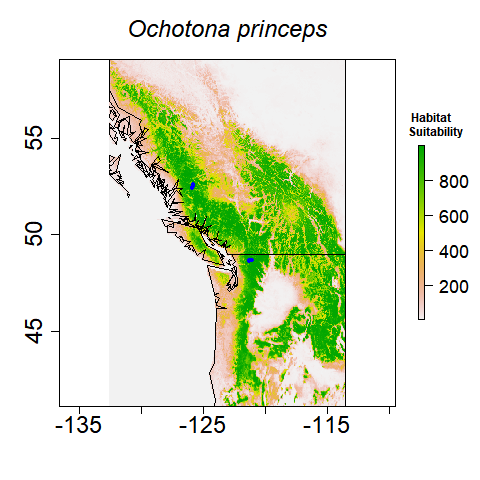

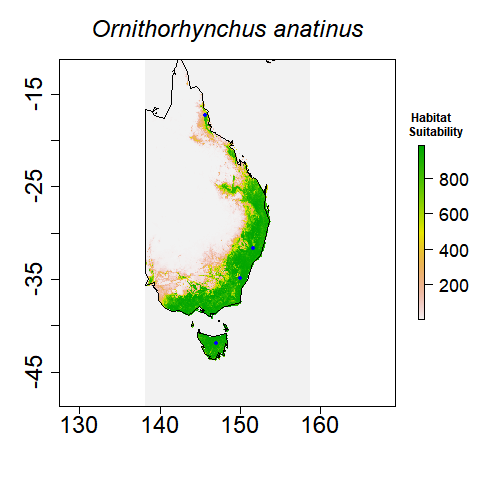

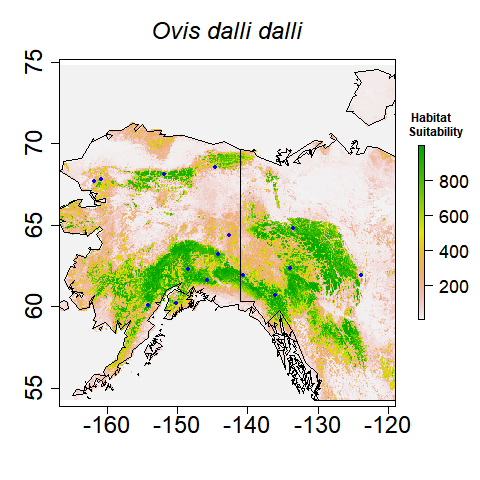

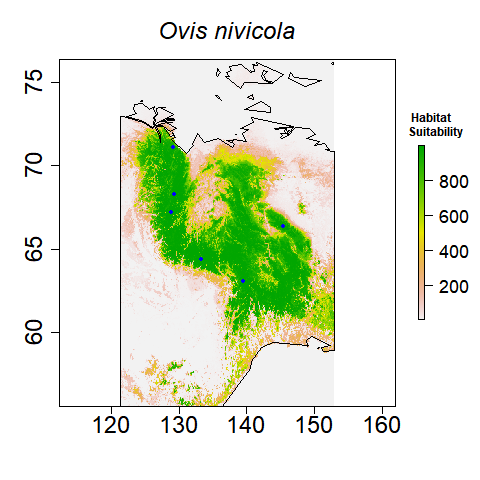

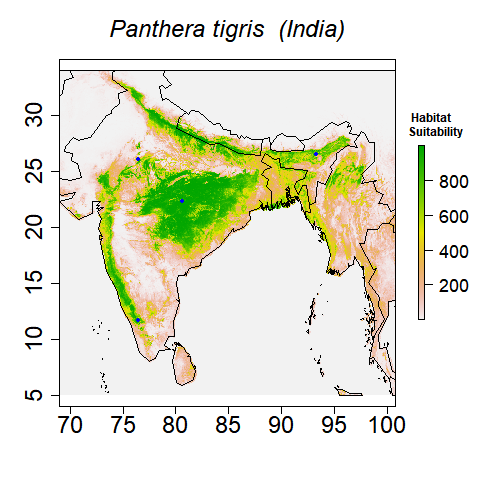

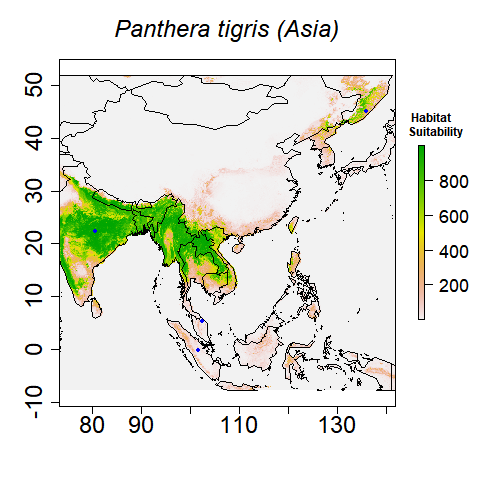

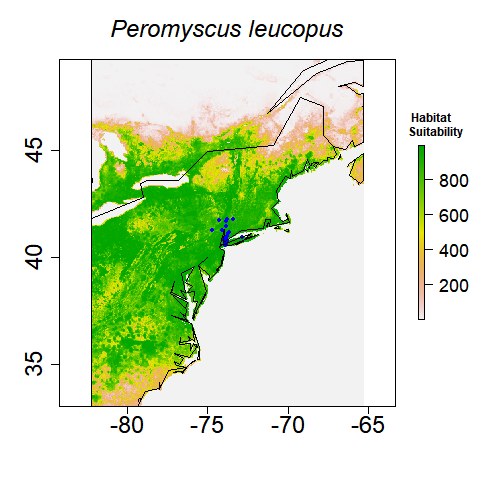

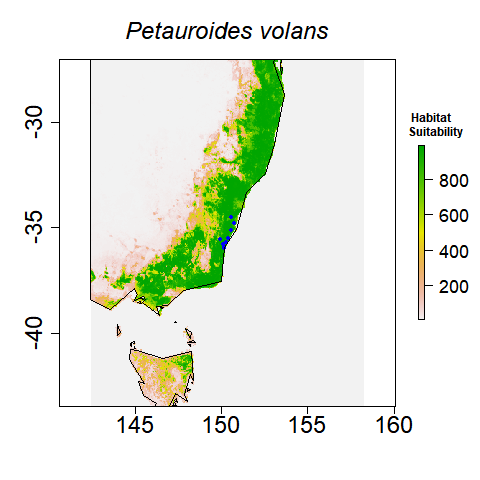

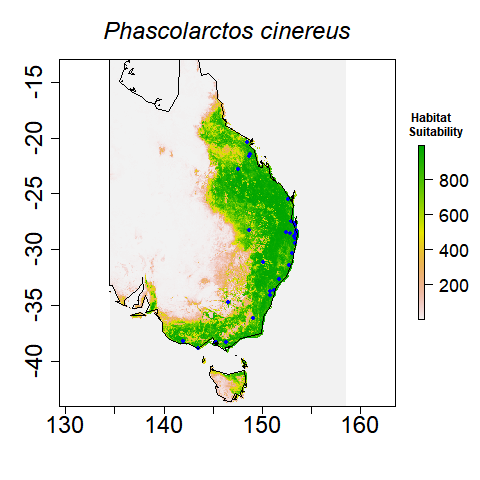

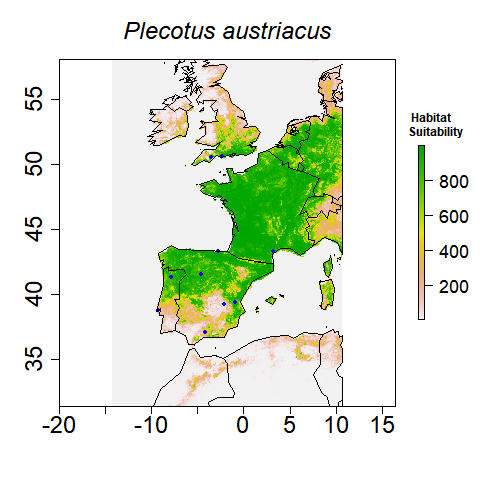

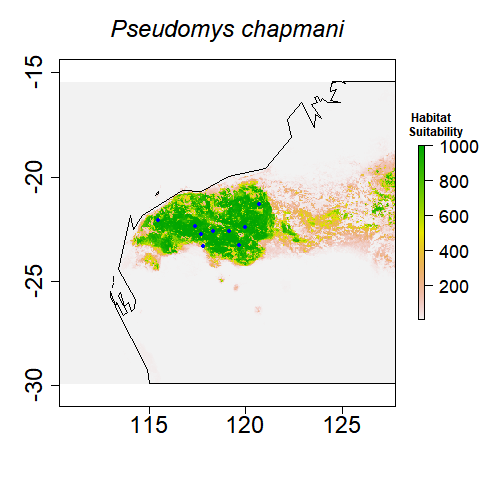

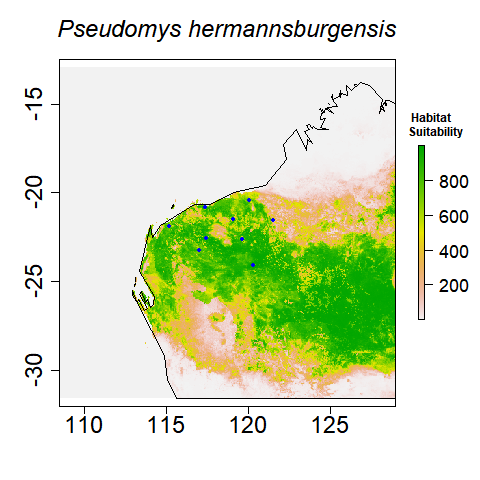

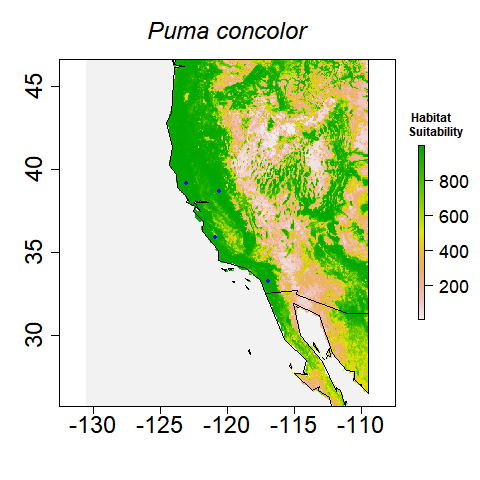

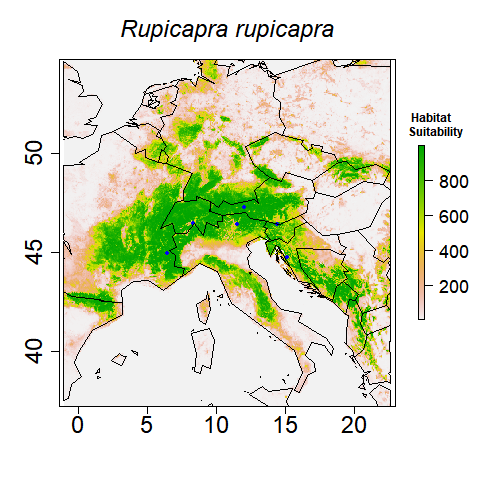

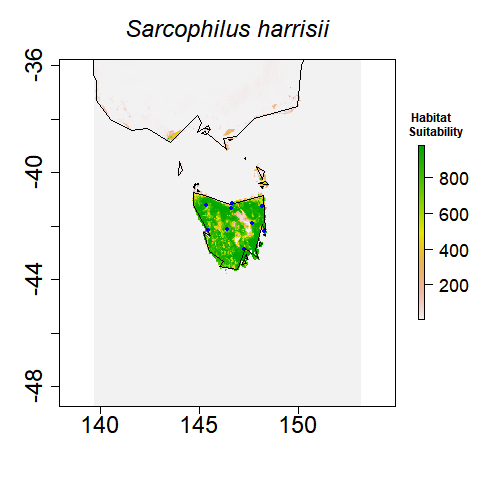

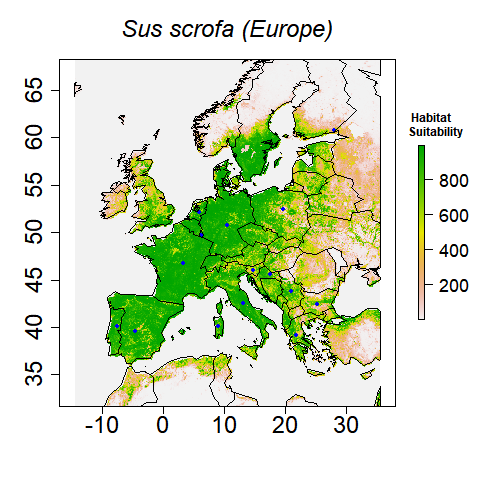

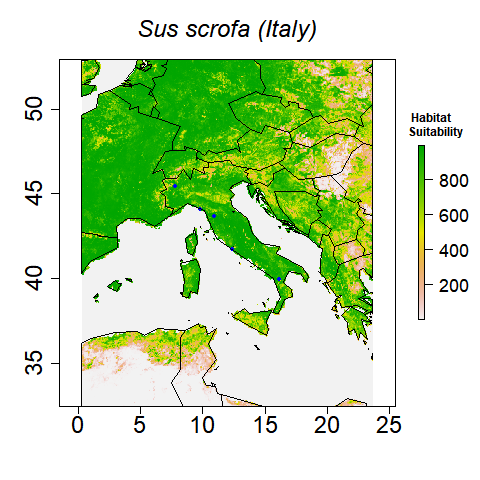

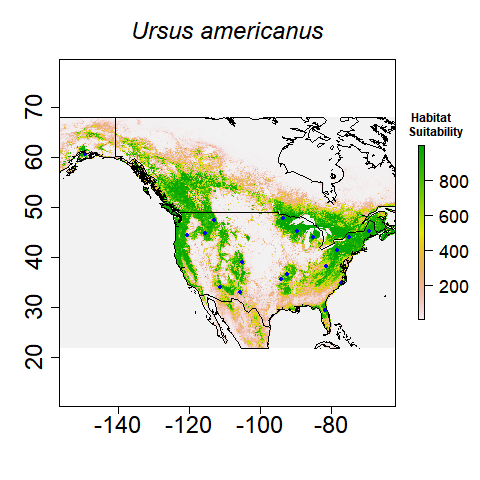

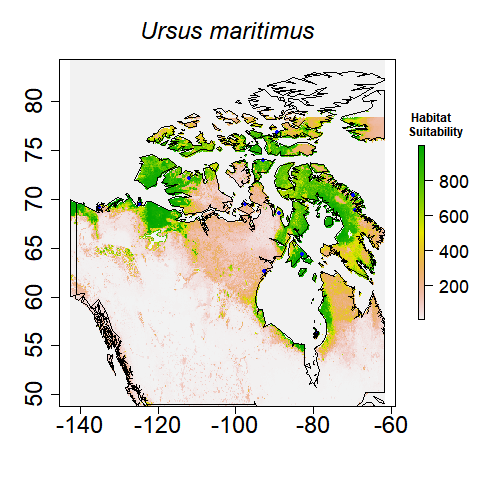

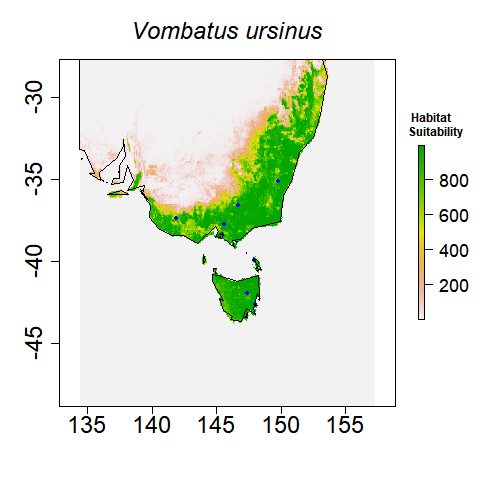

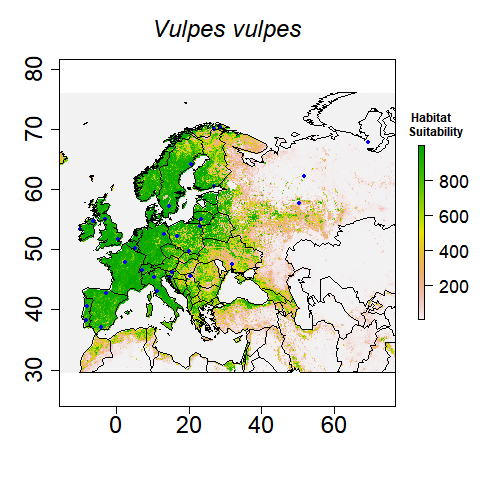

Supplement: supplementary_information_esag003 [file supplementary_information_esag003.docx]
